# Synthesis of a Novel Boronic Acid Transition State Inhibitor, MB076: A Heterocyclic Triazole Effectively Inhibits *Acinetobacter*-Derived Cephalosporinase Variants with an Expanded-Substrate Spectrum

**DOI:** 10.1021/acs.jmedchem.3c00144

**Published:** 2023-06-26

**Authors:** Rachel A. Powers, Cynthia M. June, Micah C. Fernando, Erin R. Fish, Olivia L. Maurer, Rachelle M. Baumann, Trevor J. Beardsley, Magdalena A. Taracila, Susan D. Rudin, Kristine M. Hujer, Andrea M. Hujer, Nicolò Santi, Valentina Villamil, Maria Luisa Introvigne, Fabio Prati, Emilia Caselli, Robert A. Bonomo, Bradley J. Wallar

**Affiliations:** † Department of Chemistry, Grand Valley State University, Allendale, Michigan 49401, United States; ‡ Department of Medicine, Case Western Reserve University School of Medicine, Cleveland, Ohio 44106, United States; § Research Service, Louis Stokes Cleveland Department of Veterans Affairs Medical Center, Cleveland, Ohio 44106, United States; ⊥ Pharmacology, Molecular Biology and Microbiology, Biochemistry, and Proteomics and Bioinformatics, Case Western Reserve University School of Medicine, Cleveland, Ohio 44106, United States; # CWRU-Cleveland VAMC Center for Antimicrobial Resistance and Epidemiology (Case VA CARES), Cleveland, Ohio 44106, United States; ∇ Department of Life Sciences, University of Modena and Reggio Emilia, via Campi 103, Modena 41125, Italy

## Abstract

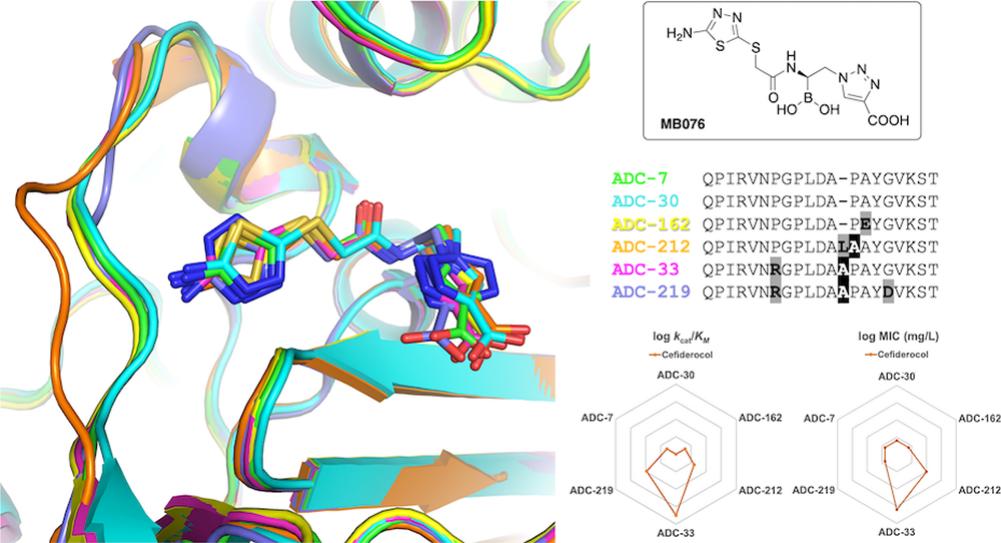

Class C *Acinetobacter*-derived cephalosporinases (ADCs) represent an important target for inhibition in the multidrug-resistant pathogen *Acinetobacter baumannii*. Many ADC variants have emerged, and characterization of their structural and functional differences is essential. Equally as important is the development of compounds that inhibit all prevalent ADCs despite these differences. The boronic acid transition state inhibitor, **MB076**, a novel heterocyclic triazole with improved plasma stability, was synthesized and inhibits seven different ADC β-lactamase variants with *K*_i_ values <1 μM. **MB076** acted synergistically in combination with multiple cephalosporins to restore susceptibility. ADC variants containing an alanine duplication in the Ω-loop, specifically ADC-33, exhibited increased activity for larger cephalosporins, such as ceftazidime, cefiderocol, and ceftolozane. X-ray crystal structures of ADC variants in this study provide a structural context for substrate profile differences and show that the inhibitor adopts a similar conformation in all ADC variants, despite small changes near their active sites.

## Introduction

*Acinetobacter baumannii* is a critical Gram-negative pathogen notable for its expanded-spectrum cephalosporin and carbapenem resistance, making it a significant challenge for clinicians to treat. The Centers for Disease Control and Prevention (CDC) recently reported that the number of cases of carbapenem-resistant *Acinetobacter* increased by ∼35% in 2020, exacerbated by the fact that hospitals had more patients who needed an extended length of stay during the COVID pandemic.^1^ Of the multiple resistance mechanisms exhibited by *Acinetobacter*, expression of β-lactamases is the most prevalent. These enzymes hydrolyze β-lactam antibiotics through destruction of the amide bond of the conserved β-lactam ring. There are four classes of β-lactamases (A, B, C, and D), with classes A, C, and D using a serine-based mechanism that involves a two-step acylation/deacylation process. The class C *Acinetobacter*-derived cephalosporinases (ADCs) play a significant role in antibiotic resistance in *A. baumannii*. β-Lactamase-mediated resistance to β-lactams can be overcome using combination therapy involving a β-lactamase inhibitor coupled with a partner β-lactam antibiotic. Boronic acids are competitive, reversible, non-β-lactam inhibitors with a long history of class C β-lactamase inhibition, making them attractive as lead compounds.^2−5^ More recently, cyclic and bicyclic boronic acids, such as vaborbactam and taniborbactam, have been approved as clinical β-lactamase inhibitors, confirming the success of boronic acids in combination therapies.^6−9^

**Figure 1:**
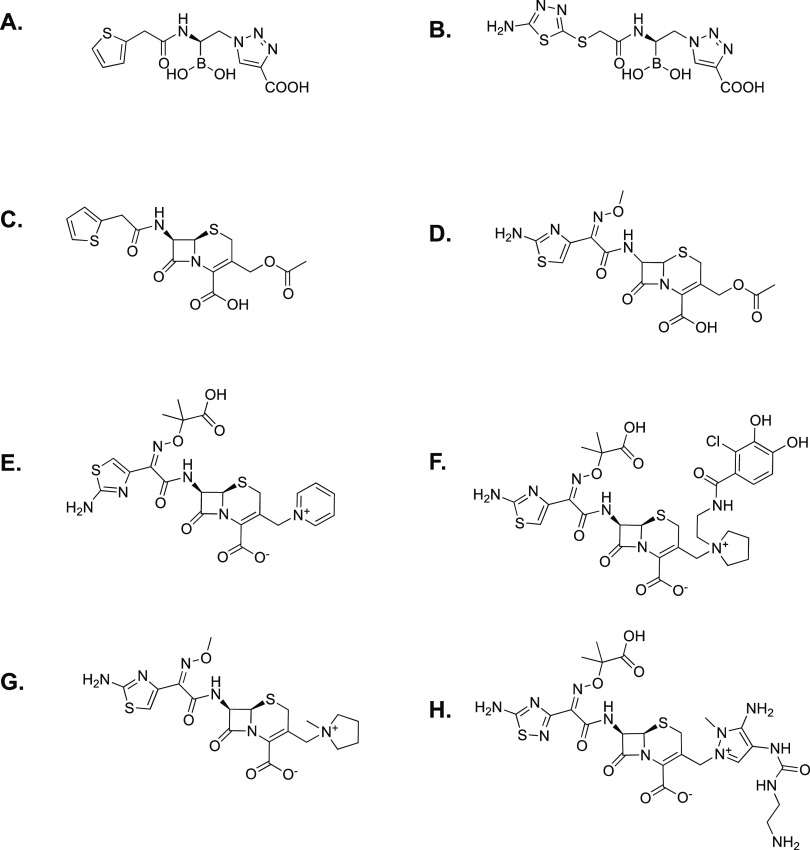
Structures of the antibiotic substrates and BATSI inhibitors used in this study: (A) **S02030**; (B) **MB076**; (C) cephalothin; (D) cefotaxime; (E) ceftazidime; (F) cefiderocol; (G) cefepime; (H) ceftolozane.

Previously, studies showed that the noncyclic boronic acid **S02030** (Figure 1) bound with high affinity to ADC-7 (*K*_i_ 44 nM) and exerted a synergistic effect against *A. baumannii* when coupled with ceftazidime.^10^ The structural analysis of this inhibitor in ADC-7 inspired further studies elucidating its activity. As a result, **S02030** was shown to be an effective β-lactamase inhibitor of KPC-2, SHV-1, MOX-1, and CTX-M variants.^11−14^**S02030** offered a compelling lead in our optimization efforts to improve the penetration and stability of these inhibitors. However, as thiophene rings have been shown to potentially lead to highly reactive metabolites,^15−17^ the analog **MB076** (patent WO2022187362) was designed and synthesized with an aminothiadiazole group hypothesized to be more stable. **MB076** was tested for activity against *A. baumannii* (Figures 1 and 3).

**Figure 2:**
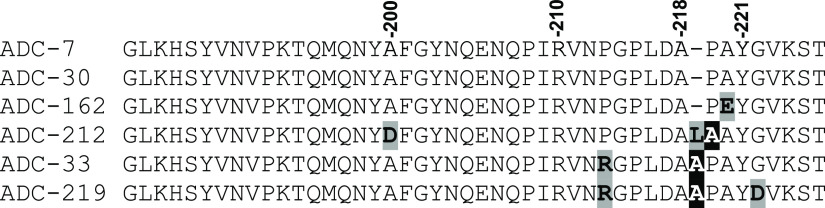
Multiple alignment of the Ω-loop region of ADCs-7, -30, -162, -212, -33, -219 using Clustal Omega.^22^ The SANC numbering scheme positions are listed above the sequences.^23^

Recently, we analyzed the profile of β-lactamases expressed in a collection of carbapenem-resistant isolates of *A. baumannii*(18) and reported the prevalence of several class C ADCs found in combination with class D OXA enzymes that provide resistance to cephalosporins and carbapenems, respectively. A wide variety of ADCs were discovered in these isolates, with ADC-30, -162, and -212 being the most prevalent, and ADC-33 and -219 only slightly less so. These enzymes share ∼99% sequence similarity, differing by only 1–3 amino acids. Many of the differences center in the Ω-loop region (residues 183–226), which has been implicated in the acquisition of an expanded spectrum of activity.^19−21^ In fact, ADC-33 was reported to hydrolyze ceftazidime and cefepime, but not carbapenems.^19^ Based on the multiple sequence alignment (Figure 2 and Supp Figure 4), several variants differ at residues that directly flank Tyr221. For example, ADC-30 and ADC-162 differ only at position 220 (Ala220 in ADC-30; Glu220 in ADC-162), and ADC-33 and -219 differ only at position 222 (Gly222 in ADC-33; Asp222 in ADC-219). Additionally, ADC-33 and -219 contain a Pro213Arg mutation and an alanine duplication a few residues prior to Tyr221. ADC-212 has a Pro219Leu mutation and also contains an alanine duplication of a couple of residues prior to Tyr221. As these three variants all contain an alanine duplication, ADC-33, -212, and -219 will be referred to collectively as the Adup variants.

With the presence of several important ADC variants circulating in *A. baumannii* isolates, we hypothesized that **MB076** would bind with high affinity and effectively inhibit all representative variants from the ADC family. In addition, we explore the questions of whether amino acid changes in the ADC variants result in functional differences that expand the substrate profile, and if so, what is the structural basis for the observed functional differences?

To specifically understand the role of the most prevalent ADCs, as well as ADC-7, we used microbiological assays, steady-state kinetics, and X-ray crystallography to characterize a novel non-β-lactam boronic acid (**MB076**) that inhibits our panel of prominent ADCs with high affinity. In addition, this combination of techniques has defined structure/function relationships of these prominent ADC β-lactamase variants and insight into how resistance to cephalosporin β-lactams evolves in *A. baumannii*.

## Results and Discussion

### Design and Synthesis of the Boronic Acid Inhibitor **MB076**

**Figure 3:**
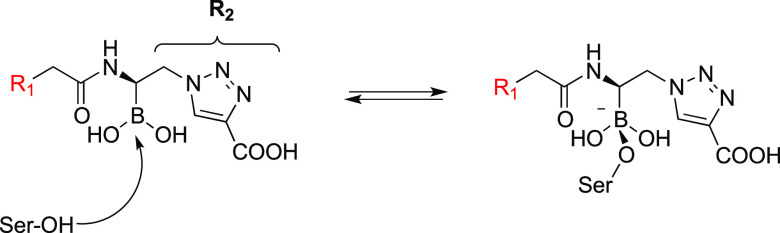
Schematic representation of the BATSIs binding to the active site of a serine β-lactamase resembling the tetrahedral transition state of the β-lactam hydrolysis reaction leading to inhibition. In the case of BATSIs, this is a reversible competitive process.

1-Amido-2-triazolylethaneboronic acid proved to be a family of chiral boronic acids active against representative β-lactamases that are found in critical pathogens.^24^ In these compounds, the phenyl ring present in previously synthesized chiral 1-amido-2-phenylethane boronic acids is substituted by a triazole ring, which confers a similar inhibitory profile (*K*_i_ values) with improved in vitro activity (MICs).^24^ In particular, **S02030**, bearing the 2-thienylacetamido side chain of the second-generation cephalosporin cephalothin (Figure 1), is an excellent inhibitor of ADC-7 (*K*_i_ = 44 nM), KPC-2 (IC_50_ = 80 nM) and SHV-1 (IC_50_ = 130 nM).^14,25^ In an attempt to maintain the effectiveness of this inhibitor while replacing the thiophene ring with a moiety that would be more stable and resistant to biological oxidation,^15−17^ we designed compound **MB076**, replacing the 2-thiophene ring with a 5-amino-1,3,4-thiadiazol-2-thiol ring (Figure 1). While earlier generations of cephalosporins, such as cephalothin and cefoxitin, contain thiophenes, the expanded-spectrum cephalosporins, which include cefiderocol, ceftazidime, and ceftolozane, have evolved to contain R1 side chains that more closely resemble the aminothiadiazole group of **MB076**. The motivation for incorporating these common heterocyclic rings into expanded-spectrum cephalosporin structures is due to their enhanced Gram-negative penetration and increased affinity for transpeptidase enzymes.^26^ Notably, we decided not to include in the structure of **MB076** the oxime group typical of more recent cephalosporins because this sterically hindered moiety is known to interact unfavorably in the β-lactamase binding site which could potentially decrease the binding affinity to the inhibitor (Figure 3).^26^

**Scheme 1:**
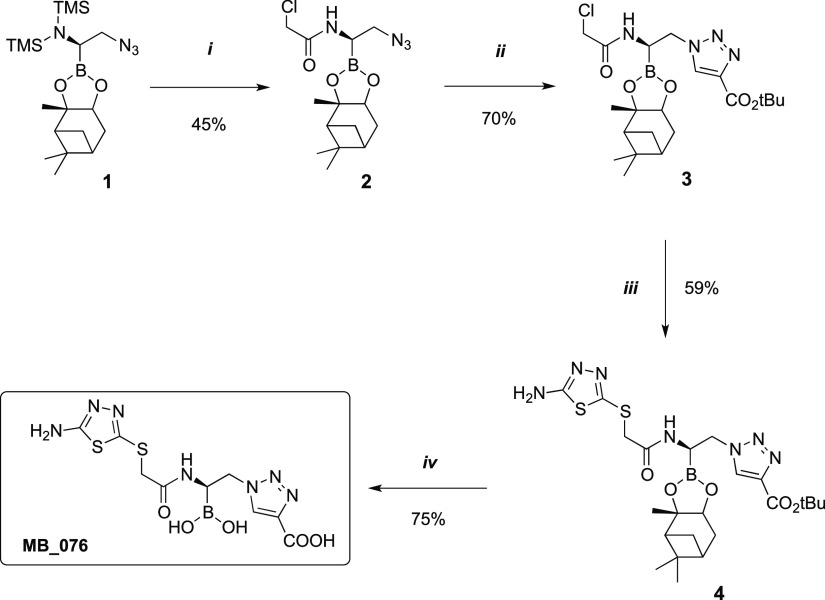
(i) (1) MeOH, THF, 0 °C to RT, (2) ClCH_2_COCl, THF, −30 °C; (ii) *t*-Butylpropiolate, Sodium Ascorbate, CuSO_4_, *t-*ButOH/H_2_O 1:1, 60 °C; (iii) 5-Amino-1,3,4-thiadiazole-2-thiol, TEA, MeCN, RT; (vi) (1) TFA, Et_3_SH, DCM, 0 °C to RT, (2) Isobutylboronic Acid, HCl 3 M, MeCN/*n-*Hexane 1:1, RT

The synthesis of **MB076**, depicted in Scheme 1, starts from 2-azido-1-*N*,*N*-bis(trimethylsilyl)amine **1**.^24^ Removal of the two TMS groups was achieved by reaction with a stoichiometric amount of methanol, and subsequent acylation of the free amine with chloroacetyl chloride led to compound **2** in 45% yield. Copper-catalyzed cycloaddition of this latter with *t-*butyl propiolate in water/*t*-butyl alcohol afforded the expected triazole **3** (70% yield), which was purified by crystallization and subjected to nucleophilic substitution with 5-amino-1,3,4-thiadiazole-2-thiol in dry acetonitrile, leading to **4** in 59% yield. Finally, deprotection of the carboxylic and boronic acids by trifluoroacetic acid in dichloromethane and i*-*butylboronic acid in acetonitrile/hexane, respectively, allowed us to obtain **MB076** in 75% yield.

### Stability of **MB076** and **S02030**

The in vitro stability of **MB076** and **S02030** was evaluated by incubating the compounds in both buffer (pH 7.4) and human plasma at 37 °C over 48 h. Half-lives (*t*_1/2_) were then calculated for each compound.

**Figure 4:**
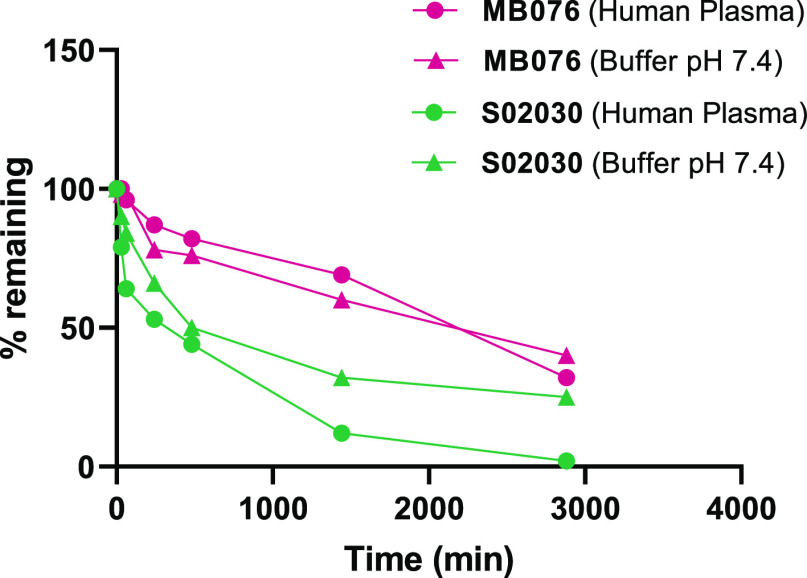
Comparison of the in vitro stability of **MB076** and **S02030** in plasma and in buffer (pH 7.4). Percentage of compound remaining over time is displayed.

Plasma and buffer stability samples were analyzed by monitoring the disappearance of the compounds using high-performance liquid chromatography-mass spectrometry (LC–MS) techniques. A highly sensitive and simple LC–MS assay was developed and validated for the quantification of **MB076** and **S02030**. The remaining percentage of both compounds versus time is presented in Figure 4. **MB076** showed excellent stability in human plasma, with a *t*_1/2_ value of 29 h, notably higher than the value obtained for **S02030** (9 h; Supplemental Figure 3). Similar results were found in buffer pH 7.4 wherein **MB076** showed a significantly longer elimination half-life with respect to **S02030** (*t*_1/2_ = 33 and 8 h, respectively; data not shown) that favors regimens with clinically relevant dosing.

### Characterization of MB076 Inhibition of ADC Variants

Inhibition kinetics demonstrates that **MB076** binds tightly to all ADC variants selected for this study and inhibits the turnover of nitrocefin (Table 1). While the variants contain a small number of amino acid residue differences, notably in the Ω-loop region, all ADCs were inhibited by **MB076** with *K*_i_ values <1 μM. The *K*_i_ of **S02030** inhibiting ADC-7 was previously reported to be 44.5 nM.^10^ The *K*_i_ values of **S02030** were also determined with each of the newer variants. Binding affinities of **S02030** were similar to those of **MB076**, with all variants possessing *K*_i_ values <1 μM, and ADC-30 having the highest affinity for both.

**Table 1:** *K*_i_ Values for ADC/BATSIs

enzyme	*K*_i_ S02030 (μM)	*K*_i_ MB076 (μM)
ADC-7	0.045 ± 0.002^a^	0.21 ± 0.016
ADC-30	0.028 ± 0.002	0.058 ± 0.005
ADC-162	0.16 ± 0.010	0.79 ± 0.039
ADC-33	0.11 ± 0.004	0.10 ± 0.004
ADC-219	0.81 ± 0.050	0.11 ± 0.019
ADC-212	0.34 ± 0.026	0.61 ± 0.038

a Ref (10).

Antimicrobial susceptibility testing (AST) of *E. coli* strains expressing the ADC variants cloned into pBCSK(−) was next performed (Tables 2 and 3). The addition of **MB076** to CAZ lowered the MICs of 3/6 isolates to ≤4 mg/L (Table 2). The three isolates that remained were ADC-33, -212, and -219, which had MICs ranging from 16 to 64 mg/L with the addition of **MB076**. The addition of **MB076** to CTX brought 5/6 isolates into the intermediate or susceptible range of CTX, with only ADC-212 retaining an MIC of 16 mg/L (Table 2). However, this still reflects a 3-fold doubling dilution reduction of the MIC. **MB076** also brought about a significant lowering of the TOL MICs by a factor of 3–4 doubling dilutions. Even for ADC-33 and ADC-212 that had the highest TOL MIC values, the MICs were reduced from 256 to 16 mg/L with the addition of **MB076**.

**Table 2:** Broth Microdilution AST Results for ADC Variants in pBCSK(−) *E. coli* DH10B in This Study^a^

β-lactamase	CAZ	CAZ + MB076	CTX	CTX + MB076	TOL	TOL + MB076	FDC	FDC + MB076	FEP
ADC-7	64	4	64	1	8	1	0.5	0.5	0.25
ADC-30	128	2	64	2	8	0.5	0.5	0.5	0.25
ADC-33	2048	16	128	1	256	16	2	0.5	0.5
ADC-162	128	4	64	2	16	1	0.5	0.25	0.25
ADC-212	512	64	128	16	256	16	1	0.5	0.5
ADC-219	256	16	16	2	128	8	1	0.5	0.12

a MICs in mg/L. Antimicrobial susceptibility tests were interpreted according to 2021 CLSI criteria for *Enterobacterales*: for ceftazidime (CAZ) and cefiderocol (FDC), MIC ≤ 4 mg/L is susceptible (*S*), MIC = 8 mg/L is intermediate (*I*), and MIC ≥ 16 mg/L is resistant (*R*); for cefotaxime (CTX) MIC ≤ 1 mg/L is *S*, MIC = 2 mg/L is *I*, and MIC ≥ 4 mg/L is *R*; for ceftolozane (TOL), no CLSI breakpoints have been defined. Most isolates were highly susceptible to cefepime (FEP, ≤0.25 mg/L, *S*) and were therefore not tested with **MB076**. **MB076** was used at a fixed concentration of 10 mg/L.

**Table 3:** Agar Dilution AST Results for the ADC Variants in pBCSK(−) *E. coli* DH10B for CAZ + MB076, CAZ + S02030, and CAZ + VAB^a^

β-lactamase	CAZ	CAZ + MB076	CAZ + S02030	CAZ + VAB
ADC-7	64	2	4	32
ADC-30	64	2	2	32
ADC-33	1024	16	128	512
ADC-162	128	8	4	128
ADC-212	256	32	32	256
ADC-219	64	4	16	32

a MICs in μg/mL. **MB076**, **S02030**, and **VAB** held at a fixed concentration of 10 mg/L. Antimicrobial susceptibility tests were interpreted according to 2021 CLSI criteria for *Enterobacterales*: for ceftazidime (CAZ), MIC ≤ 4 mg/L is susceptible (*S*), MIC = 8 mg/L is intermediate (*I*), and MIC ≥ 16 mg/L is resistant (*R*); for ceftazidime/vaborbactam (CAZ/VAB), no CLSI breakpoints have been defined. However, meropenem/vaborbactam (MEM/VAB), MIC ≤ 4/8 mg/L is *S*, MIC = 8/8 mg/L is *I*, and MIC ≥16/8 mg/L is *R*, can be used as an interpretive guideline.

We next compared the commercially available BATSI vaborbactam (VAB) to **MB076** and **S02030** using 10 mg/L of each. **MB076** compared favorably against **S02030** and VAB; it lowered the CAZ MICs for all ADC variants by 3–4 doubling dilutions lower than did VAB at the same concentration (Table 3). Interestingly for the ADC-33 variant, **MB076** increased susceptibility to CAZ 3-fold better than **S02030**.

Therefore, consistent with the ability of **MB076** to bind and inhibit the ADC variants, this BATSI brought about increased susceptibility of the *E. coli* DH10B *bla*_ADC_ variants to ceftazidime, cefotaxime, and ceftolozane, as well as being more effective than CAZ/VAB, and equal to or better than **S02030** in comparison.

To assess the structural basis for inhibition by **MB076**, the X-ray crystal structures of ADC-7 and each of the variants were determined in complex with **MB076** to resolutions ranging from 1.21–1.83 Å (Suppl Table 1). Initial *F*_o_–*F*_c_ electron density maps (contoured at 3σ) indicated the presence of the **MB076** inhibitor bound in the active sites of each of the ADC enzymes and allowed for the entire inhibitor to be modeled. For clarity, the SANC system is used for residue numbering throughout. Continuous electron density was observed between the catalytic Ser64Oγ and the boron atom of the BATSI, suggesting the dative covalent bond that is formed with these transition-state analog inhibitors. Polder omit maps confirmed the conformation of the BATSI in the active sites of the final models (Suppl Figure 6).

**Figure 5:**
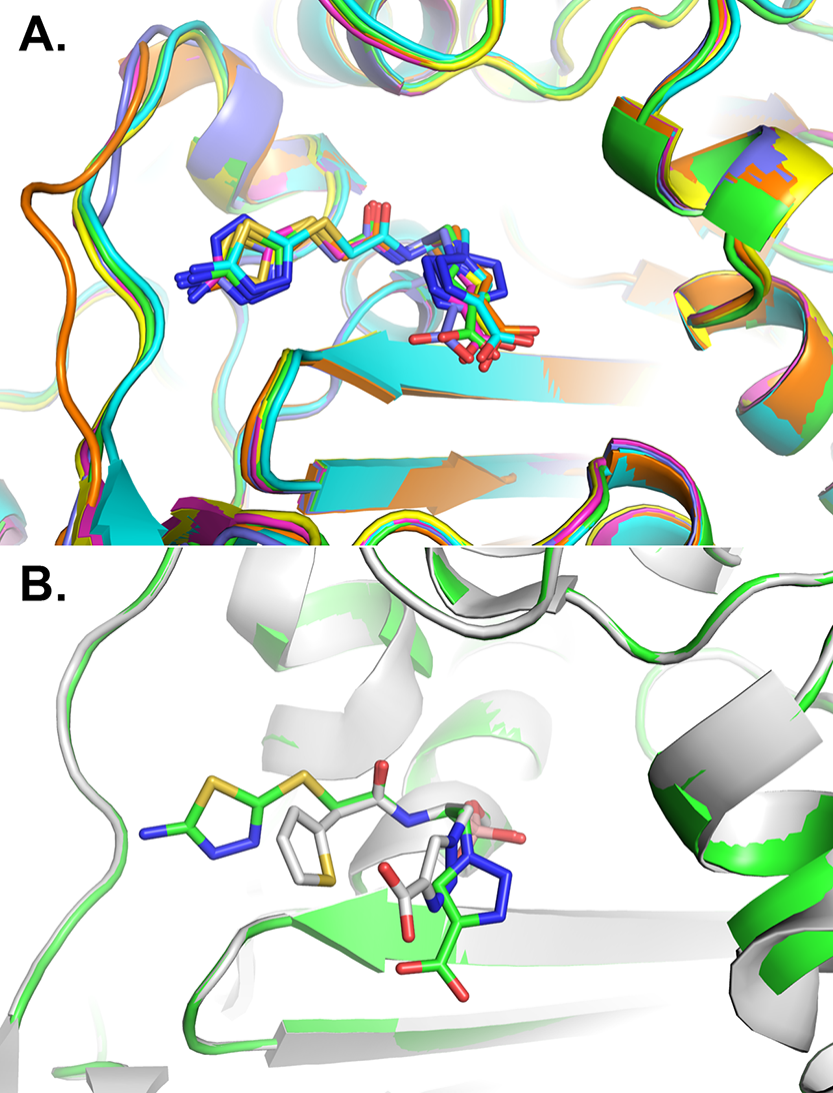
(A) Superposition of ADCs in complex with the BATSI **MB076**. Carbons for each enzyme are colored as follows: ADC-7 (green, 8FQM), ADC-30 (cyan, 8FQW), ADC-33 (magenta, 8FQO), ADC-162 (yellow, 8FQQ), ADC-212 (orange, 8FQS), and ADC-219 (purple, 8FQU). Nitrogen atoms are blue, oxygens red, and sulfurs yellow. The Ω-loop is shown on the left-hand side of the image, near the distal aminothiadiazole ring of the R1 group of **MB076**. (B) Superposition of ADC-7 bound to lead compound **S02030** (carbons colored white) with its complex with **MB076**. This figure and all subsequent crystallographic structure images were created with PyMOL (Schrodinger, LLC).

**Figure 6:**
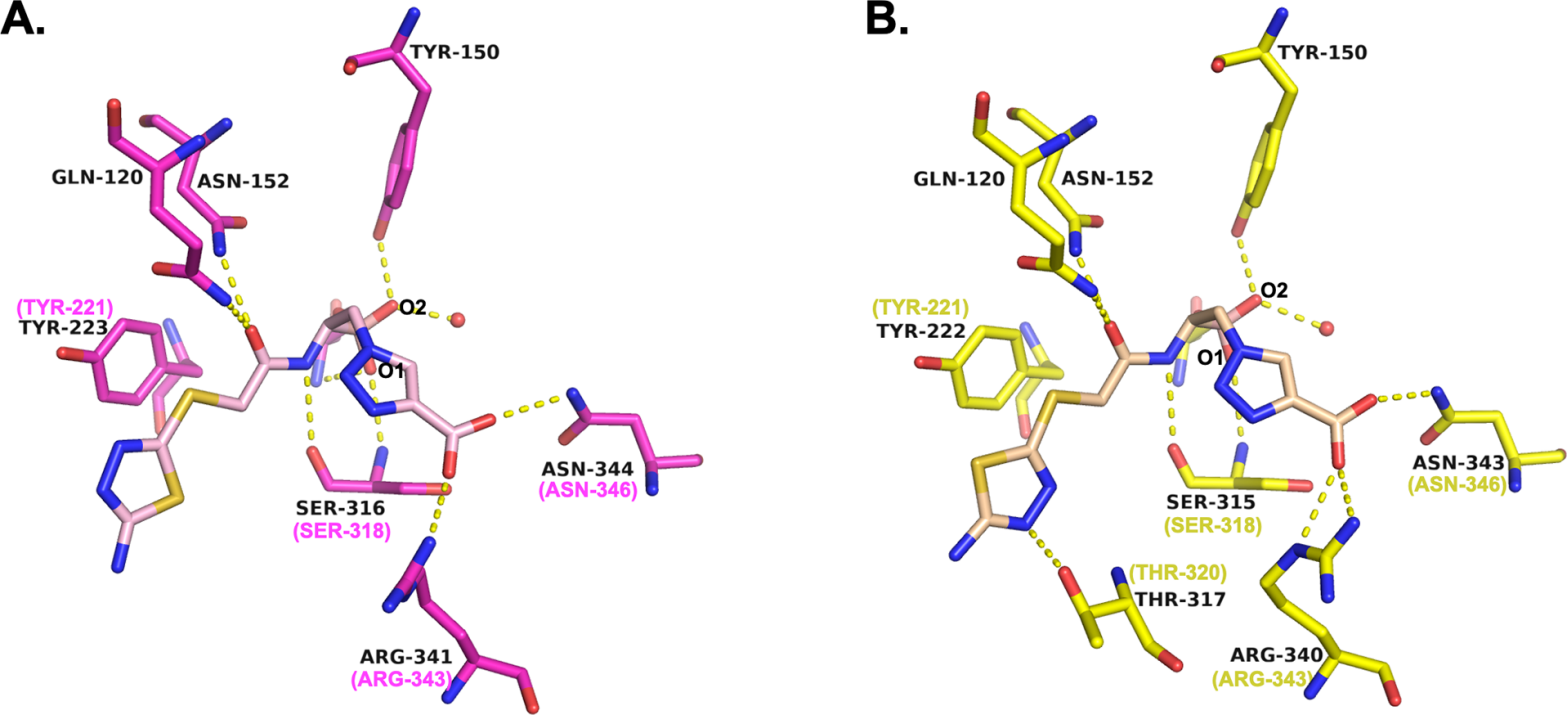
Conserved interactions between representative ADCs and **MB076**. The active sites of (A) ADC-33 (carbons magenta, 8FQO) and (B) ADC-162 (carbons yellow, 8FQQ). Carbon atoms of the inhibitor are colored light pink (ADC-33) and light peach (ADC-162) for contrast. Where SANC numbering differs from the PDB residue numbering, the SANC number is indicated in parentheses and magenta or yellow. Hydrogen bonding interactions are indicated with yellow dashed lines for distances between 2.5 and 3.2 Å. Water molecules are drawn as red spheres.

The inhibitor binds to each of the ADCs in a similar conformation (Figure 5A), maintaining key canonical interactions observed in other β-lactamase/BATSI complexes (Figure 6). The boronic acid moiety adopts the tetrahedral geometry formed by these transition-state analogs. The O1 hydroxyl group is bound in the oxyanion hole making hydrogen bonds with the main chain nitrogens of the catalytic serine (Ser64) and Ser318. The O2 hydroxyl forms hydrogen bonds with the side chain hydroxyl of Tyr150 and a water molecule that is commonly observed in BATSI complexes with class C β-lactamases.^27,28^ The O2 group is believed to represent the position of the deacylating water molecule in the transition state, and the crystallographic water molecule hydrogen bonded to the O2 atom suggests the direction of approach of the deacylating water molecule. Taken together, this supports the observed tetrahedral structure of the complex with **MB076** resembling the deacylation transition state. **MB076** contains both an R1 and R2 group (Figure 3) intended to resemble the β-lactam substrates that also contain functional groups at these positions. The R1 amide oxygens of **MB076** make hydrogen bonding interactions with the side chains of conserved amide recognition residues Gln120 and Asn152, and the R1 amide nitrogen interacts with the main chain carbonyl oxygen of Ser318, similar to those made between class C β-lactamases and β-lactams. On the other side of the inhibitor, the R2 group orients the carboxylate group into a carboxylate binding region composed of Arg343 and Asn346. The carboxylate group of **MB076** shows the greatest positional variability among the complexes. However, Arg343 is also observed in different conformations, suggesting its flexibility in inhibitor recognition. The only minor difference between the complexes is in the ADC-33 complex, where the thiadiazole ring of the R1 group in this structure is rotated ∼180° from the others. The predominant conformation of the ring is present in the final model; however, weak electron density suggests the possibility of a low occupancy alternate conformation that would be the same as the other variant complexes with **MB076**.

Comparison of the **MB076** complexes with the structure of ADC-7 bound to lead compound **S02030** (PDB 4U0X) showed that the canonical interactions with the boronic moiety and the R1 amide group are the same between the two inhibitors, but **S02030** adopts a more compact conformation in the active site, with the heterocyclic rings of the R1 and R2 groups of **S02030** forming favorable intramolecular interactions (Figure 5B). This conformation results in slightly different positions of the R2 group and its carboxylate, as well as the R1 thiophene ring. In contrast, the R1 aminothiadiazole group of **MB076** extends toward the Ω-loop, with the amino groups forming a hydrogen bond with the main chain of residue 212 (in ADC-30, -162, -219) and the nitrogens of the thiadiazole ring forming hydrogen bonds with main chain or side chain atoms of residue 320 in the β5/β6 loops of all the ADCs, except ADC-33.

**Figure 7:**
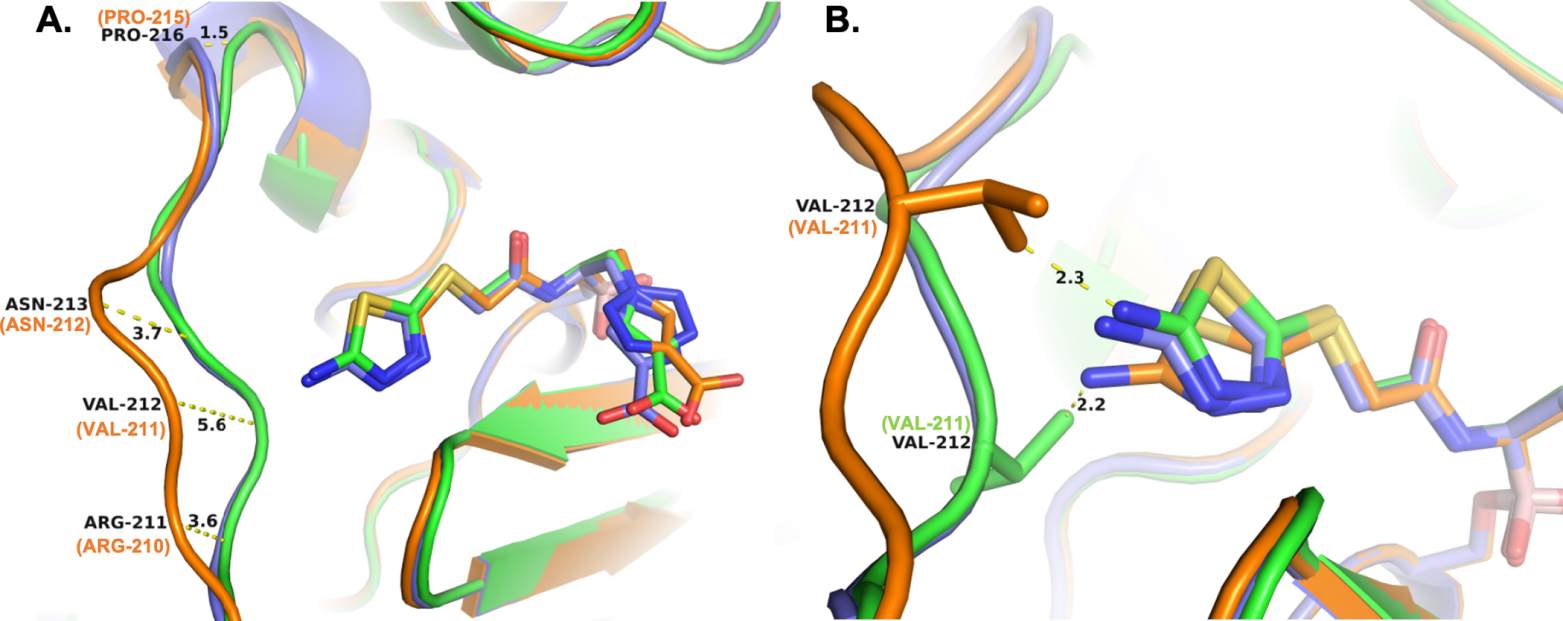
(A) Changes in the trajectory of the Ω-loop in ADC-212 (orange, 8FQS) and ADC-219 (purple, 8FQU). The conformation of the Ω-loop in ADC-7 (green, 8FQM) is shown for comparison, representing the predominant loop structure in the other variants. ADC-212 (B monomer) and ADC-219 (A monomer) were superposed with ADC-7 (B monomer) with RMSDs in Cα positions of 0.294 Å and 0.288 Å, respectively. (B) Presumed clash with Ω-loop residue Val211 of ADC-212 results in a slightly different conformation for MB076. Where SANC numbering differs from the PDB residue numbering, the SANC number is indicated in parentheses and orange or green.

Despite the residue differences between the variants, the conformations of the Ω-loop regions are generally the same in the **MB076** complexes. Two exceptions are ADC-212 and ADC-219 (orange and purple, respectively; Figure 7A) where the Ω-loop follows different trajectories. Using ADC-7 for comparison, the loop is the most altered in ADC-212, with Cα shifts ranging from 3.6 to 5.6 Å for residues 210–212. In general, the Ω-loop of ADC-212 extends away from the active site and the bound inhibitor. However, the side chain of Val211 is oriented such that it would clash with the amino group of **MB076** as observed in the other conformations (2.3 Å; Figure 7B). As a result, for ADC-212, the thiadiazole ring rotates ∼30° to avoid a clash with Val211. This conformation is not possible in the other complexes, as the amino group would clash with Val211 found in the other Ω-loops (2.2 Å). In ADC-219, the most substantial Cα shift occurs at residue 215 (1.5 Å). ADC-212 also resembles ADC-219 in this region. The shift at residue 215 is less drastic than at the other positions in ADC-212 but is still noticeably different from the rest.

### Structure/Function Effects of ADC Variants

**Figure 8:**
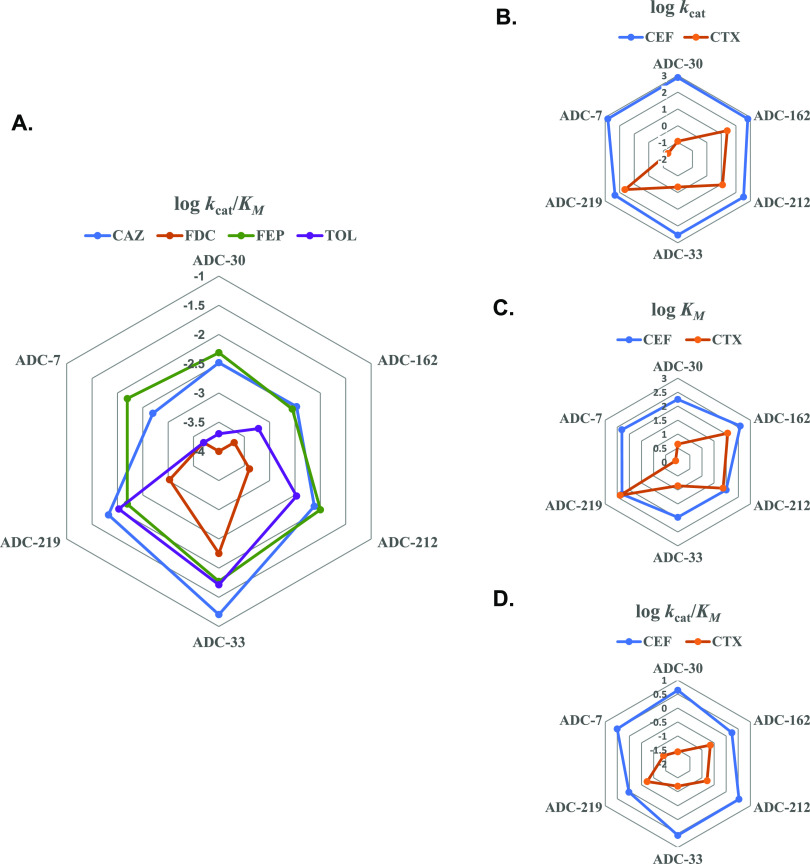
Radar plots of kinetic parameters for the ADC variants with various cephalosporin substrates. (A) *k*_cat_/*K*_M_ for ADC variants with ceftazidime (CAZ), cefiderocol (FDC), ceftolozane (TOL), and cefepime (FEP). (B) *k*_cat_, (C) *K*_M_, and(D) *k*_cat_/*K*_M_ for ADC variants with cephalothin (CEF) and cefotaxime (CTX). Values from Table 4 are plotted as logarithms.

The boronic acid **MB076** displays submicromolar *K*_i_ values for all ADC variants, yet these ADCs exhibit important differences in their ability to bind and turn over cephalosporins (Table 4 and Figure 8). In order to test a range of cephalosporin substrates and relative side chain interactions and sizes, the panel consisted of first-generation cephalothin, third-generation cefotaxime and ceftazidime, and the fourth- and fifth-generation cefepime, ceftolozane, and cefiderocol (Figure 1). Along with a bulky R2 side chain, ceftazidime, cefiderocol, and ceftolozane all have the same R1 side chain that contains the carboxydimethyloxyimino group, both of which contribute to making them large cephalosporins.

**Table 4:** Kinetic Parameters for ADC Variants

enzyme and substrate	*K*_M_ (μM)	*k*_cat_ (s^–1^)	*k*_cat_/*K*_M_ (μM^–1^/s^–1^)^a^
ADC-7			
nitrocefin	57.5 ± 4.6	720.0 ± 18.6	12.52 ± 1.33
cephalothin	204.2 ± 11.2	659.0 ± 16.6	3.22 ± 0.26
cefotaxime	1.23 ± 0.17	0.0475 ± 0.0012	0.0386 ± 0.0063
cefepime	>500		*0.0064*
ceftazidime	>500		*0.0020*
cefiderocol	>500		<*0.001*
ceftolozane	>500		<*0.001*
ADC-30			
nitrocefin	46.3 ± 3.7	538.0 ± 12.9	11.62 ± 1.21
cephalothin	174.8 ± 8.8	763.0 ± 16.5	4.36 ± 0.31
cefotaxime	4.35 ± 1.81	0.117 ± 0.009	0.027 ± 0.013
cefepime	>500		*0.0049*
ceftazidime	>500		*0.0033*
cefiderocol	>500		<*0.001*
ceftolozane	>500		<*0.001*
ADC-162			
nitrocefin	88.1 ± 8.06	591.0 ± 20.0	6.71 ± 0.84
cephalothin	382.0 ± 26.4	656.5 ± 22.8	1.72 ± 0.18
cefotaxime	115.2 ± 15.5	25.4 ± 1.4	0.220 ± 0.042
cefepime	>500		*0.0028*
ceftazidime	>500		*0.0034*
cefiderocol	>500		<*0.001*
ceftolozane	>500		<*0.001*
ADC-33			
nitrocefin	45.8 ± 6.07	707.0 ± 28.2	15.44 ± 2.66
cephalothin	95.3 ± 3.5	347.2 ± 4.5	3.64 ± 0.18
cefotaxime	7.11 ± 1.05	0.463 ± 0.014	0.063 ± 0.010
cefepime	>500		*0.0169*
ceftazidime	59.3 ± 7.80	3.71 ± 0.088	0.0626 ± 0.0097 *(0.0473)*
cefiderocol	107.2 ± 13.8	0.6003 ± 0.0315	0.0056 ± 0.0010 *(0.0053)*
ceftolozane	219.3 ± 21.6	4.23 ± 0.18	0.0193 ± 0.0027 *(0.0181)*
ADC-219			
nitrocefin	33.8 ± 3.69	270.0 ± 8.1	7.99 ± 1.11
cephalothin	200.3 ± 14.7	211.2 ± 7.3	1.05 ± 0.11
cefotaxime	238 ± 14.9	44.2 ± 0.29	0.186 ± 0.013 *(0.190)*
cefepime	>500		*0.0064*
ceftazidime	>500		*0.015*
cefiderocol	>500		*0.0010*
ceftolozane	>500		*0.0094*
ADC-212			
nitrocefin	59.2 ± 6.33	724.0 ± 25.3	12.23 ± 1.74
cephalothin	99.7 ± 5.20	337.0 ± 6.3	3.38 ± 0.24
cefotaxime	74.2 ± 18.9	12.0 ± 0.91	0.162 ± 0.054
cefepime	>500		*0.0100*
ceftazidime	>500		*0.0076*
cefiderocol	>500		<*0.001*
ceftolozane	>500		*0.0034*

a *k*_cat_/*K*_M_ values in italics were calculated from linear fits of *v*_o_ in low [*S*] ranges (<0.08 × *K*_M_).

For cephalothin, the Adup variants exhibited approximately 2-fold slower turnover (*k*_cat_ values ∼211 to 347 s^–1^) than ADC-7, -30, and -162 (*k*_cat_ values ∼657–763 s^–1^). However, ADC-33 and ADC-212 exhibited at least 2-fold lower *K*_M_ values (95.3, 99.7 μM) than ADC-7, -30, and-162 (175–382 μM). Due to the higher *K*_M_ and the lower *k*_cat_, ADC-7, -30, and-162 had the lowest *k*_cat_/*K*_M_ values (Table 4 and Figure 8D). With cefotaxime, ADC-7, -30, and -33 all bound the substrate tightly (*K*_M_ ∼ 1.23, 4.35, 7.11 μM) with slow, inversely proportional turnover numbers (*k*_cat_ 0.048, 0.12, 0.46 s^–1^). ADC-212, -162, and -219 showed a similar trend, albeit with higher *K*_M_ and *k*_cat_ values. For cefotaxime, ADC-212, -162, and -219 had *K*_M_ values of 74.2, 115, 238 μM, respectively, with much faster inversely proportional turnover numbers (*k*_cat_ 12.0, 25.4, 44.2 s^–1^), as well as *k*_cat_/*K*_M_ values (Figure 8B–D). ADC-162 does not contain an alanine duplication, but ADC-162 and -219 both contain amino acids with carboxylate side chains adjacent to Tyr221 (ADC-162 Ala220Glu, ADC-219 Gly222Asp). These two variants show an increase in catalytic efficiency with cefotaxime, but lower *k*_cat_/*K*_M_ values for cephalothin.

For the larger cephalosporins (ceftazidime, cefiderocol, and ceftolozane), there was a notable trend for the Adup variants to have higher *k*_cat_/*K*_M_ values than ADC-7, -30, and -162 (Figure 8A). For ceftazidime, the Adup variants had increased activity, most notably ADC-33 and ADC-219 with *k*_cat_/*K*_M_ values of 0.063 and 0.015 μM^–1^ s^–1^, respectively. With *k*_cat_ = 3.71 s^–1^ and *K*_M_ = 59.3 μM for ADC-33, these values agree with previously published work.^19^ Among all variants, ADC-33 also has the highest catalytic efficiency for cefepime (*k*_cat_/*K*_M_ = 0.017 μM^–1^ s^–1^), although all ADCs show very poor affinity to cefepime (*K*_M_ > 500 μM). In the case of the other cephalosporins, cefiderocol and ceftolozane, ADC-33 gained the ability to bind and turn them over (cefiderocol: *k*_cat_ = 0.60 s^–1^, *K*_M_ = 107.2 μM; ceftolozane: *k*_cat_ = 4.23 s^–1^, *K*_M_ = 219.3 μM). ADC-219 shows the next highest catalytic efficiencies, but the *K*_M_ values for ADC-219 for these substrates are higher than ADC-33 (*K*_M_ > 500 μM). For the three larger cephalosporins (ceftazidime, cefiderocol, and ceftolozane), ADC-33 had the highest ability to bind the antibiotic substrates (lowest *K*_M_) and overall catalytic efficiency (*k*_cat_/*K*_M_). A radar plot of the *k*_cat_/*K*_M_ values (Figure 8A) shows an overall trend of higher catalytic efficiency by the Adup variants, with the highest activity by ADC-33.

**Figure 9:**
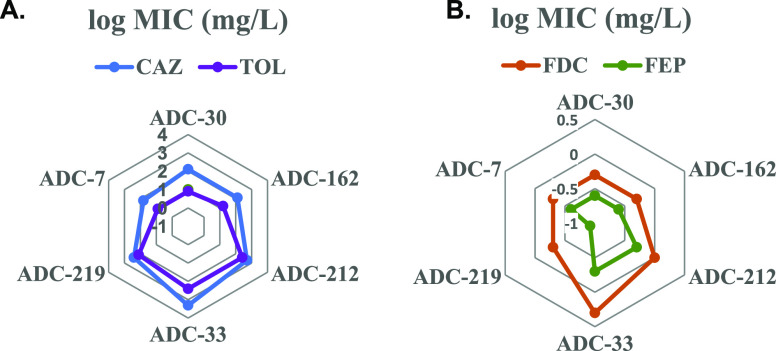
Radar plots of AST results for ADC variants. (A) MICs for ADC variants with ceftazidime (CAZ) and ceftolozane (TOL); (B) MICs for ADC variants with cefiderocol (FDC) and cefepime (FEP). Values from Table 2 are plotted as logarithms.

AST of *E. coli* strains expressing the ADC variants cloned into pBCSK was performed (Tables 2 and 3). As expected, the cephalosporin minimum inhibitory concentrations (MICs) were high for the ADC variants. ADC-33 had the highest ceftazidime (CAZ, 2048 mg/L), cefotaxime (CTX, 128 mg/L), and ceftolozane (TOL, 256 mg/L) MICs. ADC-212 also had high CAZ (512 mg/L), CTX (128 mg/L), and TOL (256 mg/L) MICs. MICs ranged from 64 to 2048 mg/L for CAZ, and 16 to 128 mg/L for CTX, with ADC-7 having the lowest overall MICs. MICs of all variants for cefiderocol (FDC) were all in the susceptible range of 0.5–2 mg/L, as were the MICs for cefepime (FEP). Radar plots of log MIC values with the larger cephalosporins (Figure 9) show the trend of the Adup ADC variants being more resistant to the larger cephalosporins, with ADC-33 being the most resistant.

**Figure 10:**
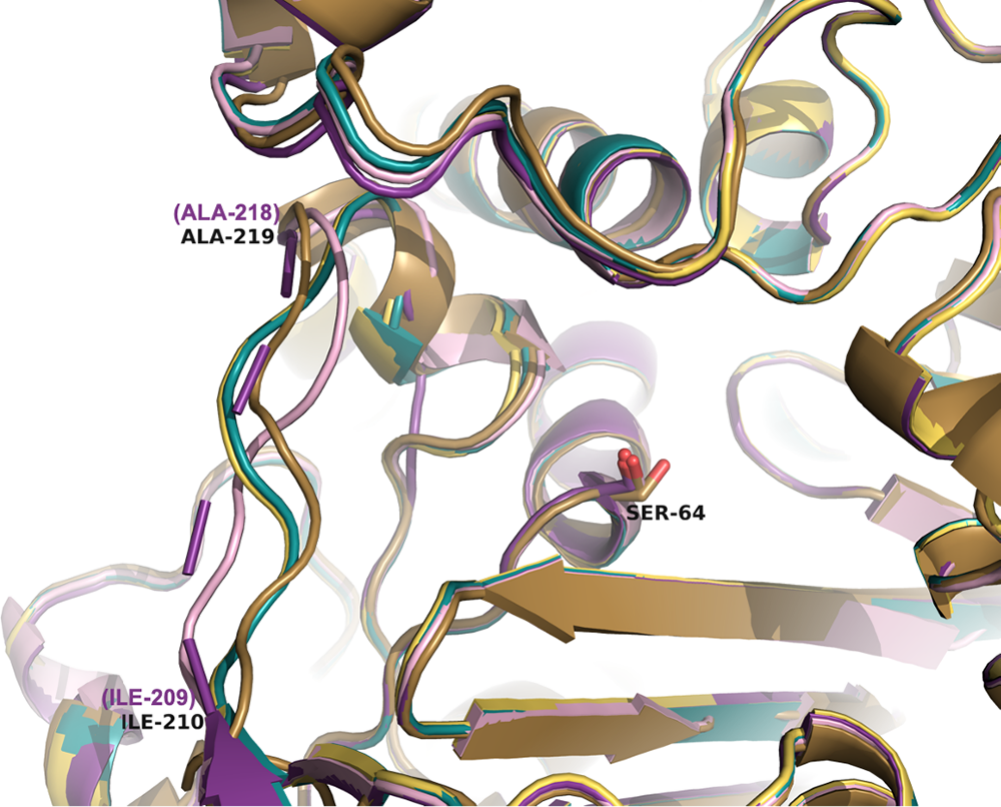
Comparison of the Ω-loop region in the apo ADC variant structures. The active site serine residue (Ser64) is labeled. Ile209 and Ala218 are labeled to indicate the area of the Ω-loop. Carbons of ADC-30 are colored turquoise (8FQV), ADC-33 are light pink (8FQN), ADC-162 light yellow (8FQP), ADC-212 gold (8FQR), and ADC-219 violet (8FQT). Where SANC numbering differs from the PDB residue numbering, the SANC number is indicated in parentheses and violet.

In the apo X-ray crystal structures of the variants (resolutions ranging from 1.24–1.89 Å), the Ω-loop conformations show more variation than in the complexes. The two variants lacking an Ala duplication in this region (ADC-30 and ADC-162) have Ω-loops with nearly identical conformations (Figure 10). Two of the variants containing an Ala duplication, ADC-33 and ADC-212, begin to diverge in their trajectories starting at Ile209, with the most significant shift in the Cα position (3.4 Å) occurring at the sequence difference between the two: Arg213 in ADC-33 vs Pro213 in ADC-212. The Ω-loops of these variants come back into alignment at Leu216. ADC-219, another variant with an Ala duplication, shows weak electron density throughout the Ω-loop, and residues 211–218 (SANC 210–217) were unable to be modeled into this region. Interestingly, ADC-33 and to a lesser extent ADC-219 are the only two variants that show the ability to hydrolyze cefiderocol. The substitution of the constrained Pro213 to an arginine in the Ω-loop, coupled with an Ala duplication may provide the flexibility necessary to accommodate this bulky cephalosporin into the active site for hydrolysis.

The area surrounding the location of the Ala duplication (residues 217–223) also exhibits structural differences between variants. In Adup variants ADC-33 and -212, residues Leu216 and Asp217 are extended out from the active site, as compared to ADC-7, -30 and -162. In contrast, ADC-30 and -162 form a tight turn that restricts the active site with the Leu216 side chain oriented toward the interior of the enzyme, and the side chain of Asp217 forming hydrogen bonds with the main chain amide nitrogen of Gly214, both presumably stabilizing this turn.

**Figure 11:**
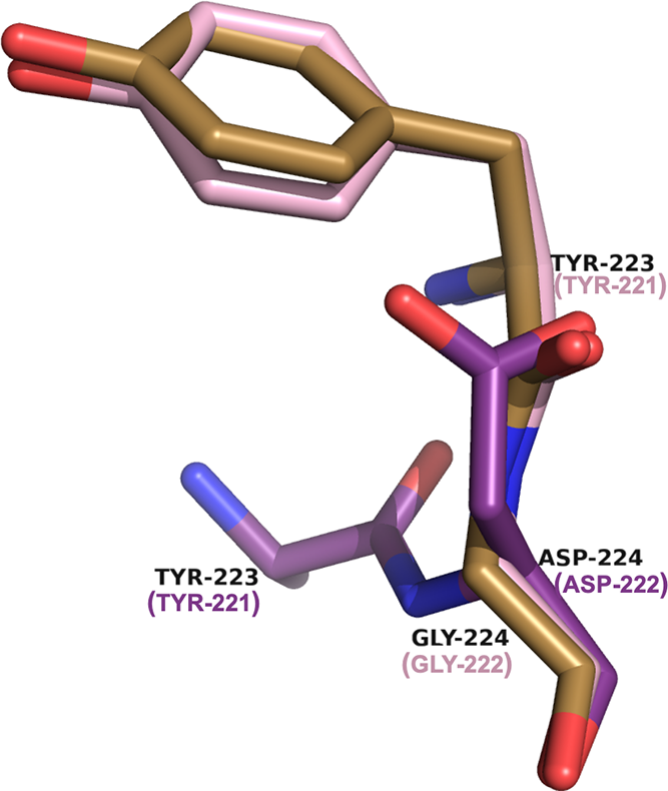
Alternate location of Tyr221 in the apo ADC-219 (violet, 8FQT) structure (side chain not visible in the electron density map). The mutation of Gly222Asp in ADC-219 repositions Asp222 into the position usually occupied by Tyr221. The standard position of Tyr221 is indicated in the apo ADC-33 (light pink, 8FQN) and apo ADC-212 (gold, 8FQR) structures for comparison. SANC numbering for residues is provided in parentheses.

Despite sequence differences at positions 218a and 219 in ADC-33 (Ala218a, Pro219) and ADC-212 (Leu218a, Ala219), the structure is nearly identical, with negligible changes in corresponding Cα positions. However, these variants display subtle but noticeable shifts in the side chain of conserved residue Tyr221, as compared to variants without the Ala insertion. Perhaps most interesting is the structure of ADC-219 in this region. The Ala duplication (Ala218, Ala218a) of ADC-219 are the first residues observed after the completely disordered region (unmodeled residues 210–217) in this enzyme, and the alanines are out of register with those in ADC-33 and -212, with Ala218 of ADC-219 overlaying with Pro215 of ADC-33 and -212. The most striking downstream consequence of this shift in ADC-219 is that Tyr221 is not observed in its standard orientation forming the base of the active site of the class C enzymes. Instead, Tyr221 is reoriented approximately 5 Å from the position in ADC-33, as measured between Cα atoms, although electron density for the Tyr side chain is not observed. This shift of Tyr221 results in the side chain of Asp222 occupying the space left vacant by the tyrosine residue (Figure 11). Whereas most of the sequence differences between the variants occur prior to Tyr221, ADC-219 is the only variant to contain a sequence difference after it (Figure 2). The higher *B*-factors in this region of ADC-219, as well as its weaker electron density as compared to the other structures, suggest a more flexible, mobile loop that potentially samples multiple conformations. A similar drastic movement in the position of Tyr221 was observed in the apo structure of the expanded-spectrum variant of the class C β-lactamase from *Enterobacter cloacae* GC1.^29^

**Figure 12:**
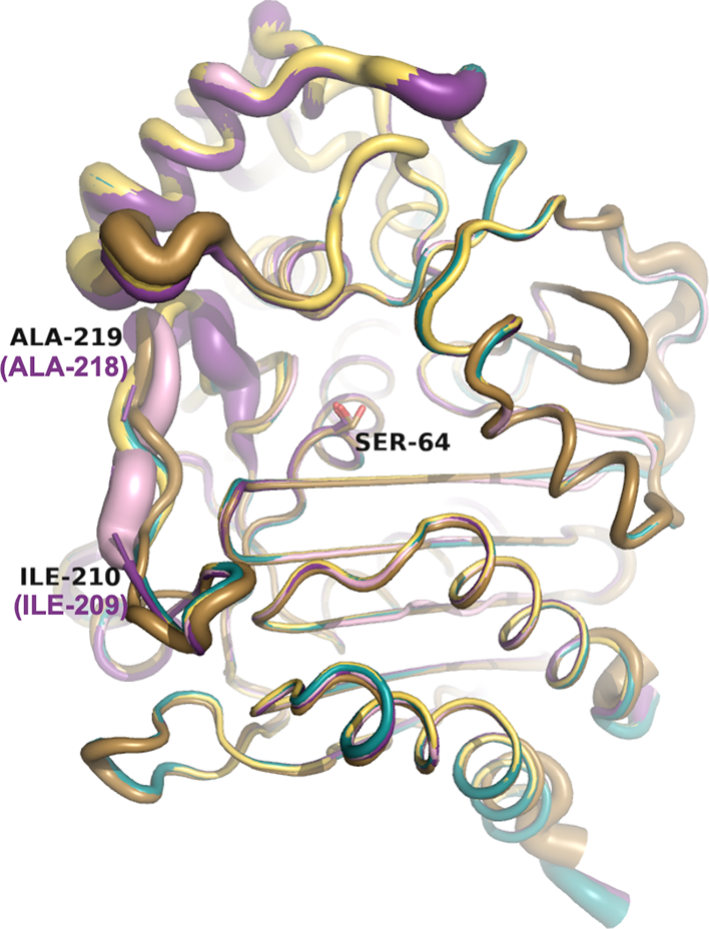
Analysis of *B*-factors for the variants in their apo forms. The Ω-loop contains higher *B*-factors as indicated by the larger tubes, suggesting increased mobility. Carbons of ADC-30 are colored turquoise (8FQV), ADC-33 are light pink (8FQN), ADC-162 light yellow (8FQP), ADC-212 gold (8FQR), and ADC-219 violet (8FQT). Where SANC numbering differs from the PDB residue numbering, the SANC number is indicated in parentheses and violet.

Given the observed differences in the Ω-loop trajectories, as well as the sequence differences in this region, the *B*-factors of the final models of the apo structures were analyzed. For each individual ADC variant, the average overall *B*-factors for all protein atoms was directly compared to the average *B*-factors of the atoms in the Ω-loops (residues 183–226) of the corresponding monomer (Suppl Table 1). Overall, the *B*-factors of atoms in the Ω-loops are elevated more in the Adup variants, suggesting that the Ω-loop is more flexible in this region of the Adup variants: ADC-30 (loop 20.4 Å^2^; B monomer 21.0 Å^2^) and ADC-162 (loop 23.0 Å^2^; B monomer 22.3 Å^2^) vs ADC-33 (loop 20.8 Å^2^; B monomer 18.8 Å^2^) ADC-212 (loop 21.8 Å^2^, B monomer 19.7 Å^2^) ADC-219 (loop 41.4 Å^2^, B monomer 35.7 Å^2^). Interestingly, the structure of the Ω-loop appears to impact the conformation of a loop that sits “above” it (residues 122–127 on which Gln120 is found), as this region also shows elevated *B*-factors when comparing the variants. Overall, the highest *B*-factors occur in the Ω-loops of ADC-33 (light pink) and ADC-219 (violet), as indicated by the larger tubes (Figure 12).

### Superpositions of Ω Loops in apo vs Complexes

**Figure 13:**
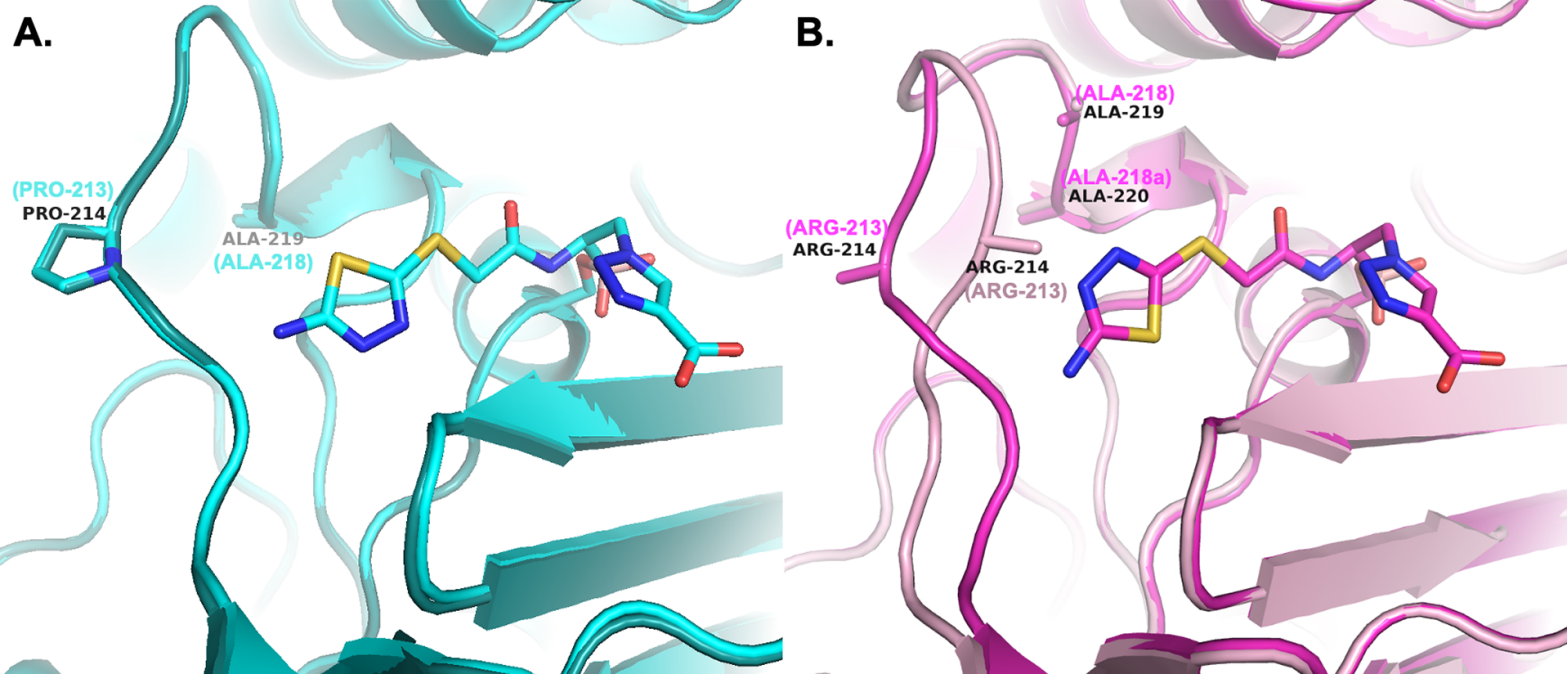
Comparison of the Ω-loops upon **MB076** inhibitor binding. (A) Variants that do not contain an Ala insertion; ADC-30 (apo and complex) is shown as a representative example. (B) Ala insertion variants (Adup); ADC-33 (apo-light pink and complex-magenta) as a representative example. The side chain of Arg213 was not visible in the electron density maps of either the apo or complexed structures of ADC-33 and is indicated only by the Cβ atom of the side chain. Where SANC numbering differs from the PDB residue numbering, the SANC number is indicated in parentheses and cyan (ADC-30, 8FQV, 8FQW), light pink (ADC-33 apo, 8FQN), or magenta (ADC-33 complex, 8FQO).

Finally, comparisons were made between the apo and complexed structures of each variant. Overall, the variants that do not contain an Ala insertion showed little to no change in their Ω-loop conformations upon binding of the inhibitor **MB076** (Figure 13). In contrast, the Adup variants all showed major reorganization of their Ω-loops upon binding to **MB076**. In the ADC-33 complex, residues Arg210 and Val211 shift toward **MB076**, whereas residues Arg213, Gly214, and Pro215 shift away from **MB076** (Cα shifts 2.1–4.0 Å). In ADC-212, residues Arg210, Val211, Asn212, and Pro213 all shift away from **MB076** (Cα shifts 2.0–5.4 Å). In ADC-219, this region was disordered (residues 210–217) in the apo structure, and the entire loop becomes ordered in the **MB076** complex, positioning Tyr221 into its standard location at the base of the active site.

## Conclusions

The boronic acid transition state inhibitor, **MB076**, was synthesized in an effort to inhibit multiple class C β-lactamases. Among a set of prevalent ADC variants that contain amino acid changes near the Ω-loop region**, MB076** binds and inhibits these β-lactamases with *K*_i_ values <1 μM. The X-ray crystal structures of the ADC variants in complex with **MB076** showed that the inhibitor adopts a similar conformation in all the active sites, making the expected interactions observed between class C β-lactamases and BATSIs, despite altered Ω-loop structures in ADC-212 and ADC-219. In *E. coli* strains expressing the ADC variants, **MB076** caused increased susceptibility to ceftazidime, cefotaxime, and ceftolozane, as well as being more effective than vaborbactam with regards to ceftazidime susceptibility, which is consistent with the kinetics results. In addition, certain ADC variants exhibited increased ability to turn over larger cephalosporins, such as cefiderocol, cefepime, and ceftolozane. These ADC variants all contain an alanine duplication in the Ω-loop, with ADC-33 having the greatest ability to bind and turn over large cephalosporins, specifically cefiderocol. In contrast to the related class C β-lactamase AmpC from *E. coli*, the R2 site in the ADC variants is more open due to rearrangement of the helix containing Asn289 that orients the side chain of this residue out of the R2 site. This expanded R2 site could better accommodate larger cephalosporins into the active site for binding and catalysis, but since all the variants are similar in this region, this cannot account for the observed kinetic differences. ADC-33 is the only variant that has the ability to bind (*K*_M_ 107 μM) and hydrolyze cefiderocol, albeit slowly (*k*_cat_ 0.60 s^–1^). The greatest variability is observed in the Ω-loop of ADC-33 (Figure 13B), where the loop appears to become more flexible, likely due to the replacement of a rigid proline with an arginine at residue 213. This substitution, coupled with the alanine duplication near the R1 binding site, may allow for easier entry of these larger cephalosporins into the active site to facilitate hydrolysis. Other groups have noted similar rearrangements in regions flanking the R1 site that result in their acquired expanded-spectrum cephalosporinase activity. The class A KPC-4 double variant (Pro104Arg/Val240Gly) causes flexibility in the Ω-loop that allows the general base Glu166 back into position to facilitate hydrolysis of ceftazidime.^30^ Additionally, amino acid insertions such as an alanine duplication in the β5−β6 loop of class D OXA enzymes^31^ and the tripeptide insertion in the Ω-loop of the class C β-lactamase from *Enterobacter cloacae* GC1^29^ also result in enzymes that are better able to bind and turnover the larger cephalosporins. Consistent with the kinetics results, the Adup variants (ADC-33, -212, -219) expressed in *E. coli* DH10B also conferred higher MICs to ceftazidime, ceftolozane, cefiderocol, and cefepime. Notably, both kinetics and ASTs demonstrated that ADC-33 had the greatest capability to bind, inactivate, and decrease susceptibility to larger cephalosporins, such as cefiderocol. The structural and biochemical insights made herein provide a unique opportunity to further refine and improve synthetic efforts in designing novel compounds in the future. Further studies are warranted to define the complete spectrum of inhibitory activity of **MB076**. Microbiological studies against different isolates/strains and animal studies are underway to determine the microbiological and pharmacokinetic/pharmacodynamic properties.

## Experimental Section

### Synthesis

#### General Procedure

All reactions were performed under argon using oven-dried glassware and dry solvents. Dry tetrahydrofuran (THF) was obtained by standard methods and freshly distilled under argon from sodium benzophenone ketyl prior to use. Reactions were monitored by using thin layer chromatography (TLC) by means of Macherey-Nagel silica gel 0.20 mm (60-F_254_) under UV light (*l* = 254 nm) or developed with standard stain solution: KMnO_4_, ninhydrin, curcumin, cerium ammonium molybdate (Hanessian’s Stain) followed by heating. Chromatographic purification and isolation of the compounds was performed on gravimetric silica gel (particle size 0.05–0.20 mm). ^1^H and ^13^C NMR spectra were recorded on a Bruker Avance-400 MHz spectrometer. Chemical shifts (δ) are reported in ppm and were calibrated to the residual signals of the deuterated solvent (CDCl_3_, CD_3_OD). ^13^C NMR were recorded with ^1^H broadband decoupling. Multiplicity is given as s = singlet, d = doublet, t = triplet, q = quartet, m = multiplet, br = broad signal; coupling constants (*J*) are given in Hz. Two-dimensional NMR techniques (COSY, HMBC, HSQC) were used to aid in the assignment of signals in ^1^H and ^13^C spectra. In particular, the signal of the boron-bearitheg carbon atom in the ^13^C spectra tends to be broadened, and the signal is often beyond the detection limit, but its resonance was unambiguously determined by HSQC and HMBC. Mass spectra were determined on an Agilent Technologies LC–MS (*n*) Ion Trap 6310A (ESI, 70 eV). High-resolution mass spectra were recorded on a LC–MS apparatus: Thermo Scientific UHPLC Ultimate 3000 coupled with Q Exactive Hybrid Quadrupole-Orbitrap Mass Spectrometer. Melting points were measured in open capillary tubes on a Stuart SMP30 Melting Point apparatus. Optical rotations were determined at +20 °C on a PerkinElmer 241 polarimeter and are expressed in 10^–1^ deg cm^2^ g^–1^.

Mass spectra were determined on an Agilent Technologies LC–MS (*n*) Ion Trap 6310A (ESI, 70 eV). High-resolution mass spectra were recorded on an Agilent Technologies 6520 Accurate-Mass Q-TOF LC/MS. The purity of **MB076** was above 95%, determined by analytical HPLC-MS (see the Supporting Information for a detailed description).

##### (+)-Pinanediol (1*R*)-2-Azido-1-(2-chloroacetamido)ethylboronate (**2**)

Compound **1**(24) (4.04 mmol, 1.65 g, 1 eq) was dissolved in dry THF (40 mL) and cooled at 0 °C under argon. Dry CH_3_OH (4.04 mmol, 163 μL, 1 eq) was added, and the reaction mixture was magnetically stirred 15 min at 0 °C and then 1 h at r.t. The mixture was then cooled at −30 °C and chloroacetyl chloride (4.44 mmol, 354 μL, 1.1 eq) dissolved in dry THF (1 mL) was added over 5 min. The solution was stirred for 1 h at −30 °C, and then the mixture was diluted with ethyl acetate and quenched with saturated NaHCO_3_ aqueous solution and the phases separated. The aqueous phase was extracted three times with ethyl acetate, and the combined organic phases were washed once with a 1:1 mixture of water and brine and once with brine, dried (Na_2_SO_4_), filtered, and concentrated in vacuo to give a crude residue which was purified by two sequential column chromatography purifications on silica gel: the first was eluted with petroleum ether/ethyl acetate 7:3, the second with DCM/Et_2_O 8:2. Compound **2** was isolated as a yellow oil (620 mg, 45% yield).

^1^H NMR (400 MHz, CDCl_3_) δ: 0.85 (s, 3H, C*H*_3_), 1.27 (d, *J* = 12.4 Hz, 1H, C*H*_2_), 1.30 (s, 3H, C*H*_3_), 1.42 (s, 3H, C*H*_3_), 1.81–1.98 (m, 2H, pinanyl C*H*_2_), 2.06 (t, *J* = 5.5 Hz, 1H, pinanyl C*H*), 2.18–2.41 (m, 2H, pinanyl C*H*_2_), 3.33–3.43 (m, 1H, BC*H*), 3.54 (dd, *J* = 12.6, 7.3 Hz, 1H, BCHC*H*_2_), 3.71 (dd, *J* = 12.6, 3.7 Hz, 1H, BCHC*H*_2_), 4.10 (s, 2H, ClC*H*_2_), 4.35 (dd, *J* = 8.8, 2.1 Hz, 1H, pinanyl OC*H*), 7.09 (s, 1H, N*H*).

^13^C NMR (101 MHz, CDCl_3_) δ: 24.2, 26.4, 27.2, 28.7, 35.6, 38.3, 38.4 (B*C*H), 39.6, 42.1 (Cl*C*H_2_), 51.5, 53.0 (BCH*C*H_2_), 78.5, 86.9, 167.5 (*C*=O).

^11^B NMR (128 MHz, CDCl_3_) δ: 29.7.

HRMS [M – H]^−^: calc. for C_14_H_21_BClN_4_O_3_ 339,1401, found: 339,1405.

[α]_D_^25^ – 10.6° (*c* = 1.8, CHCl_3_).

##### (+)-Pinanediol (*R*)-(2-(4-(*tert*-Butoxycarbonyl)-1*H*-1,2,3-triazol-1-yl)-1-(2-chloroacetamido)ethyl)boronate (**3**)

CuSO_4_ (42 mg, 264 μmol, 0.1 eq) and sodium ascorbate (105 mg, 0.528 μmol, 0.2 eq) were subsequently added to degassed water (25 mL) under Ar, and the mixture was stirred for 2 min until a yellow precipitate was formed. Compound **2** (2.64 mmol, 900 mg, 1 eq) was dissolved in 25 mL of degassed *t-*BuOH and added to the mixture. *tert*-Butyl propiolate (3.96 mmol, 0.5 g, 1.5 eq) was finally added one portion via a syringe and the reaction mixture was stirred for 3 h at 40 °C. The solution was partitioned between ethyl acetate and water, and the aqueous phase was extracted three times with ethyl acetate. The combined organic phases were washed twice with brine, then dried (Na_2_SO_4_), filtered, and concentrated in vacuo to give the crude product which was purified by crystallization from Et_2_O/Pentane 1:1 to afford **3** as a yellow solid (863 mg, 70% yield). M.p.: 79–80 °C.

^1^H NMR (400 MHz, CDCl_3_) δ: 0.85 (s, 3H, pinanyl C*H*_3_), 1.26 (d, *J* = 10.3 Hz, 1H, pinanyl C*H*_2_), 1.29 (s, 3H, pinanyl C*H*_3_), 1.41 (s, 3H, pinanyl C*H*_3_), 1.60 (s, 9H, O^*t-*^Bu), 1.80–1.95 (m, 2H, pinanyl C*H*_2_), 2.04 (t, *J* = 5.5 Hz, 1H, pinanyl C*H*), 2.15–2.40 (m, 2H, pinanyl C*H*_2_), 3.61–3.71 (m, 1H, BC*H*), 4.09 (s, 2H, ClC*H*_2_), 4.36 (dd, *J* = 8.8, 2.2 Hz, 1H, pinanyl OC*H*), 4.57 (dd, *J* = 14.2, 8.1 Hz, 1H, BCHC*H*_2_), 4.68 (dd, *J* = 14.2, 4.0 Hz, 1H, BCHC*H*_2_), 7.51 (s, 1H, N*H*), 7.99 (s, 1H, triazolyl C*H*).

^13^C NMR (101 MHz, CDCl_3_) δ: 24.2, 26.5, 27.2, 28.4, 28.7, 35.7, 38.4, 39.3 (B*C*H), 39.7, 41.5 (Cl*C*H_2_), 51.5 (BCH*C*H_2_), 51.6, 78.4, 82.6, 86.7, 128.3 (triazolyl *C*H), 141.42 (triazolyl *C*), 159.9 (*C*OO^*t-*^Bu), 168.7 (HN*C*=O).

^11^B NMR (128 MHz, CDCl_3_) δ: 29.2.

HRMS [M + H]^+^ calc. for C_21_H_33_BClN_4_O_5_^+^ 467.2227, found: 467.2230.

[α]_D_^25^ – 20.8 (*c* = 1.6, CHCl_3_).

##### (+)-Pinanediol (*R*)-(1-(2-((5-Amino-1,3,4-thiadiazol-2-yl)thio)acetamido)-2-(4-(*tert*-butoxycarbonyl)-1*H*-1,2,3-triazol-1-yl)ethyl)boronate (**4**)

5-Amino-1,3,4-thiadiazole-2-thiol (2.85 mmol, 379 mg, 1.04 eq) was suspended in dry CH_3_CN (54 mL) under argon, and triethylamine (2.87 mmol, 0.4 mL, 1.05 eq) was added. The reaction mixture turned clear. Compound **3** (2.74 mmol, 1.28 g, 1 eq) was added as a solid and the reaction mixture was stirred for 6 h at r.t. The mixture was partitioned between DCM and H_2_O, and the aqueous phase was extracted four times with DCM. The combined organic phases were washed once with H_2_O, then dried (Na_2_SO_4_), filtered, and concentrated in vacuo to give the crude product which was triturated with Et_2_O and washed with hexane affording **4** as a white off solid (910 mg, 59% yield). M.p.: 79–81 °C.

^1^H NMR (400 MHz, CDCl_3_) δ: 0.86 (s, 3H, pinanyl C*H*_3_), 1.28 (s, 3H, pinanyl C*H*_3_), 1.35 (d, *J* = 10.4 Hz, 1H, pinanyl C*H*_2_), 1.41 (s, 3H, pinanyl C*H*_3_), 1.57 (s, 9H, OC(C*H*_3_)_3_) 1.75–1.94 (m, 2H, pinanyl C*H*_2_), 2.01 (t, *J* = 5.5 Hz, 1H, pinanyl C*H*), 2.09–2.42 (m, 2H, pinanyl C*H*_2_), 3.36–3.44 (m, 1H, BC*H*), 3.90 (s, 2H, SC*H*_2_), 4.27 (d, *J* = 7.6 Hz, 1H, pinanyl OC*H*), 4.54 (d, *J* = 5.7 Hz, 2H, BCHC*H*_2_), 6.95 (s, 2H, N*H*_2_), 8.06 (s, 1H, triazolyl C*H*), 9.07 (s, 1H, N*H*).

^13^C NMR (101 MHz, CDCl_3_) δ: 24.3, 26.8, 27.4, 28.4 (OC(*C*H_3_)_3_), 29.1, 33.9 (SC*H*_2_), 36.4, 38.3, 40.0, 42.2 (BC*H*), 52.1, 52.6 (BCH*C*H_2_), 77.4, 82.6, 84.9, 128.2, 140.9, 160.3 (*C*OO^*t-*^Bu), 173.2 (HN*C*=O). The two quaternary thiadiazolyl carbons were not detectable.

^11^B NMR (128 MHz, CH_3_OD) δ: 18.6.

HRMS calcd for C_23_H_34_BN_7_O_5_S_2_ + H^+^ (M + H): 564.2234, found: 564.2240.

[α]_D_^25^ – 58.1° (*c* = 1.5 in CHCl_3_).

##### (*R*)-1-(2-(2-((5-Amino-1,3,4-thiadiazol-2-yl)thio)acetamido)-2-boronoethyl)-1*H*-1,2,3-triazole-4-carboxylic Acid (MB076)

Compound **4** (1.6 mmol, 900 mg, 1 eq.) was dissolved in dry DCM (2.5 mL) and TFA (2.5 mL, approx. 20 eq.) was added with a syringe at 0 °C. The reaction mixture was stirred at r.t. for 12 h and then concentrated to dryness. DCM was added and evaporated three times to completely remove TFA, and the oily residue was used as such in the next step without further purification.

The crude product from above was dissolved in CH_3_CN (12 mL) and isobutylboronic acid (1.6 mmol, 163 mg, 1 eq.), HCl 3 M (4.76 mmol, 1.6 mL, 3 eq.) and *n*-hexane (12 mL) were sequentially added, and the resulting biphasic solution was vigorously stirred. After 20 min, the *n*-hexane solution (containing the pinanediol isobutylboronate) was removed and an equal amount of fresh *n*-hexane was added. The last procedure was repeated several times until a TLC analysis of the *n*-hexane layer did not reveal the presence of isobutylboronate. The total reaction time was 4 h. Finally, after removal of the *n*-hexane phase, the residue was concentrated to dryness. The so-obtained crude product was dissolved in CH_3_OH; addition of ethyl acetate allowed the formation of a precipitate that was filtered and triturated with CH_3_CN affording the desired inhibitor **MB076** as a light yellow solid (446 mg, 75% yield). M.p.: 84–86 °C.

^1^H NMR (400 MHz, CH_3_OD) δ: 3.27 (1H, dd, *J* = 10.6, 4.0 Hz, BC*H*), 4.14 (2H, s, SC*H*_2_), 4.43 (1H, dd, *J* = 10.6, 14.6, Hz, BCHC*H*_2_), 4.54 (1H, dd, *J* = 4.0, 14.6 Hz, BCHC*H*_2_), 8.52 (s, 1H, C*H*_triazole_).

^13^C NMR (151 MHz, CH_3_OD) δ: 33.3 (S*C*H_2_), 45.7 (B*C*H), 53.7 (BCH*C*H_2_), 130.3 (*C*H_triazole_), 141.2 (*C*_triazole_), 155.3 (*C*_thiadiazole_), 163.4 (*C*OOH), 172.7 (*C*_thiadiazole_), 175.0 (HN*C*=O) ppm.

^11^B NMR (128 MHz, CH_3_OD) δ: 15.51 ppm.

HRMS [M – H]^−^ calc. for C_9_H_12_BN_7_O_5_S_2_ 372.0362, found 372.0364.

[α]_D_^25^ – 86.1° (*c* = 3.3, CH_3_OH).

#### Expression and Purification of ADC Variants

ADC-7 β-lactamase was expressed as previously described^10^ and purified using cation exchange chromatography.^32^ The expression plasmids for the other ADCs (-30/-33/-162/-212/-219) were constructed in pET28a vectors by GenScript. For the purification of all ADCs, cell pellets were suspended in 25 mM 3-(*N*-morpholino)propanesulfonic acid (MOPS buffer), pH 6.5, with 1× HALT protease inhibitor cocktail (Sigma) and DNase I (50 Units). The solution was sonicated for 4 × 30 s intervals on ice. The lysate was centrifuged at 15,000 rpm at 4 °C for 20 min. The cell-free extract was then loaded onto a carboxymethyl-cellulose column by gravity flow at 4 °C (5 mL resin per gram of cell pellet). The column was washed with 100 mL of 25 MOPS, pH 6.5 at a flow rate of 0.3 mL/min followed by elution with a linear gradient of 0–0.5 M NaCl in 25 MOPS, pH 6.5. The fractions containing ADC were collected, pooled, and then dialyzed in 2 × 5 L of 25 MOPS, pH 6.5 at 4 °C. The dialyzed ADC was concentrated to at least 10 mg/mL using an Amicon Ultra centrifugal filter unit with Ultra-10 membrane (Millipore). The concentration of ADCs was determined using the A_280_ with an extinction coefficient of 46,300 M^–1^ cm^–1^, as calculated for the expressed residues 24–383 of all ADC variants by the ProtParam tool on the ExPASy bioinformatics portal.^33^

#### Kinetic Characterization of ADC Variants

Steady-state kinetic parameters were determined by combining pure enzyme with antibiotic substrates in 50 mM NaH_2_PO_4_, pH 7.4 at room temperature. Changes in absorbance were measured on a Cary 60 UV–Vis spectrophotometer (Agilent Technologies) and converted to velocity using the change in extinction coefficient specific to nitrocefin (ε_482_ = 17,400 M^–1^·cm^–1^), cephalothin (ε_262_ = 7660 M^–1^·cm^–1^), ceftazidime (ε_260_ = 8660 M^–1^·cm^–1^), cefotaxime (ε_260_ = 7500 M^–1^·cm^–1^), cefepime (ε_260_ = 750 M^–1·^cm^–1^), cefiderocol (ε_259_ = 9430 M^–1·^cm^–1^), and ceftolozane (ε_254_ = 6810 M^–1^·cm^–1^). Initial velocities were fit to the Michaelis–Menten equation yielding *k*_cat_ and *K*_M_ values. For ADC/substrate combinations in which the *K*_M_ was too large to accurately determine both *k*_cat_ and *K*_M_ values, the initial velocities at *k*_cat_/*K*_M_ values were calculated from linear fits of *v*_o_ in low [*S*] ranges (<0.08 × *K*_M_).

For inhibition kinetics, utilizing nitrocefin (NCF) as a colorimetric substrate, the inhibition constant (*K*_i_) of **MB076** and **S02030** BATSI with ADCs was determined using competition kinetics as previously described.^10,25,28,34^ The measurements of the initial velocities were performed with the addition of 100 μM NCF after a 3 min pre-incubation of the enzyme (2 nM) with increasing concentration of the inhibitor. To determine the average velocities (*v*_0_), data from three experiments were fit to the equation:
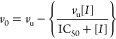
where *v*_u_ represents the NCF uninhibited velocity and IC_50_ represents the inhibitor concentration that results in a 50% reduction of *v*_u_. The *K*_i_ value was corrected for the NCF affinity (*K*_M_ values for each ADC variant listed in Table 1) with the Cheng–Prusoff^35^ equation:
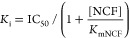


#### Crystallization and X-ray Crystal Structure Determination of ADC Variants

All ADC crystals were grown via hanging drop vapor diffusion at room temperature in 0.1 M succinate/phosphate/glycine (SPG buffer), pH 5.0, 25% w/v PEG-1500, with 3.5–3.75 mg/mL ADC enzyme as previously described.^10,25,28^ Complexes of ADC-7, -30, -33, and -162 with **MB076** were obtained by harvesting preformed crystals using a nylon loop and soaking them in crystallization buffer containing **MB076** at 5 mM for 5–80 min. For ADC-212 and -219, **MB076** was added directly to the crystallization drop to a final concentration of ∼5 mM and allowed to soak for 1 h. After soaking, crystals were harvested, flash-cooled in liquid nitrogen, and stored in pucks.

Data were measured from single crystals at the Advanced Photon Source at Argonne National Laboratory (LS-CAT 21ID-D for all data sets, except apo ADC-212 (21ID-F)). Diffraction data were processed with autoPROC,^36^ and additional processing of the structure factors was performed using STARANISO.^37^ Structures were determined by molecular replacement with Phaser^38^ using as a starting model either the structure of apo ADC-7 (PDB 4U0T) or ADC-7/**S02030** (PDB 4U0X) with all water, inhibitor, and ion atoms removed. Residues differing between the starting model and the variant were modified to match the variant sequence. The models were refined using Phenix^39,40^ followed by subsequent rounds of model building in Coot.^41^ Polder omit maps were calculated with Phenix by omitting the ligand and using a 3.0 Å solvent exclusion radius.^42^ Coordinates and structure factors are deposited with the Protein Data Bank as 8FQM (ADC-7/**MB076**), 8FQV (ADC-30 apo), 8FQW (ADC-30/**MB076**), 8FQN (ADC-33 apo), 8FQO (ADC-33 **MB076**), 8FQP (ADC-162 apo), 8FQQ (ADC-162/**MB076**), 8FQR (ADC-212 apo), 8FQS (ADC-212/**MB076**), 8FQT (ADC-219 apo), 8FQU (ADC-219/**MB076**).

#### Antimicrobial Susceptibility Testing (AST)

Susceptibility testing to standard antibiotics was performed by broth microdilution or agar dilution using an Oxoid replicator according to 2021 Clinical and Laboratory Standards Institute (CLSI) guidelines. MICs for CAZ, CTX, FEP, and TOL were determined using cation-adjusted Mueller Hinton MH broth, and MICs for FDC were done in iron-depleted cation-adjusted MH broth according to CLSI methods. **MB076**, **S02030**, and VAB were used at a fixed concentration of 10 mg/L. All MICs were interpreted according to the 2021 CLSI guidelines.^43^

#### Plasmid Constructs in pBCSK- for MIC Determinations

*bla*_ADC-7_ pBCSK- was cloned and expressed in *E. coli* DH10B cells as previously described.^44^ All other *bla*_ADC_ variants were synthesized by GenScript according to the *bla*_ADC-7_ pBCSK- strategy, cloning into the XbaI/BamHI sites of the pBCSK- vector.

#### In Vitro Stability Assays

Primary stock solutions of **MB076** and **S02030** were prepared in methanol (0.5 mM). The calibration standards were prepared from the stock solutions by diluting with methanol to concentrations of 0.5, 1, 2.5, 4, and 5 μM. The specificity of the method was evaluated as the lack of matrix interference by analysis of human drug-free plasma samples. Calibration curves were constructed in the concentration range of 0.5–5 μM, and linearity was established using least squares linear regression analysis of peak area versus nominal concentrations, and correlation coefficients (*R*^2^) higher than 0.99 were found for both **MB076** and **S02030**. The precision of the method was assessed by injecting a 5 μM solution five times, and % RSD values up to 2.0% were found. Compound recovery was evaluated by comparing the analyte peak area of the previously inactivated human plasma samples with standard solutions in methanol at equivalent concentrations and expressed as percentages. The recovery values were 92% for **MB076** and 89% for **S02030**.

##### Human Plasma Stability Assays

For the preparation of the standard solutions, human plasma was inactivated with MeOH. Then, 0.1 M phosphate buffer pH 7.4 and a solution of the compound in DMSO (2.5 μM) was added. The solutions were vortexed, filtered, and analyzed by LC–MS (*A*_*t*=0_). Samples were prepared as follows. A solution of the compound in DMSO (2.5 μM) was incubated in human plasma and 0.1 M phosphate buffer pH 7.4. The solutions were incubated at 37 °C, and at suitable time intervals, the reaction was stopped by the addition of MeOH. Solutions were vortexed and filtered. Degradation time courses were followed by LC–MS enabling the quantitation of compounds (Supplemental Figure 3). The percentage of compound remaining was calculated by area/area percentage, according to the following equation:
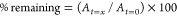
where *A*_*t*=0_ corresponds to the peak area of the standard.

Half-lives (*t*_1/2_) were calculated in Origin using a one-phase decay model with *t*_1/2_ = ln(2)/*b*, where *b* is the slope of a linear plot of natural logarithm (ln) of the remaining compound concentration (*C*) versus incubation time. Each condition was tested in triplicate.

##### Buffer pH 7.4 Stability Assays

Standard solutions were prepared by adding the compound in DMSO (2.5 μM) to 0.1 M phosphate buffer pH 7.4 and MeOH. Solutions were vortexed, filtered, and analyzed by LC–MS (*A*_*t*=0_). Samples were then prepared by adding a solution of the compound in DMSO (2.5 μM) to phosphate buffer pH 7.4 and incubating at 37 °C. At suitable time intervals, the reaction was stopped by the addition of MeOH. The solutions were vortexed and filtered. Degradation time courses were followed by LC–MS enabling the quantitation of compounds. Half-lives (*t*_1/2_) were calculated in Origin using a one-phase decay model with *t*_1/2_ = ln(2)/*b*, where *b* is the slope of a linear plot of natural logarithm (ln) of the remaining compound concentration (*C*) versus incubation time. Each condition was tested in triplicate.

## Supporting Information Available

The SMILES string for **MB076**: OB([C@H](CN1C=C(C(O)=O)N=N1)NC(CSC2=NN=C(*N*)S2)=O)O. Purity determination by HPLC trace and ^1^H, ^13^C-NMR spectra, additional figures illustrating the multiple alignment of ADCs, an overview of the entire structure of ADC-7 highlighting amino acid residues that differ from the other variants, ADC/**MB076** Polder omit maps, and a table of crystallographic statistics (PDF)

## Accession Codes

PDB codes: 8FQM (ADC-7/**MB076**), 8FQV (ADC-30 apo), 8FQW (ADC-30/**MB076**), 8FQN (ADC-33 apo), 8FQO (ADC-33/**MB076**), 8FQP (ADC-162 apo), 8FQQ (ADC-162/**MB076**), 8FQR (ADC-212 apo), 8FQS (ADC-212/**MB076**), 8FQT (ADC-219 apo), 8FQU (ADC-219/**MB076**).

## Abbreviations

ADC: *Acinetobacter*-derived cephalosporinase
AST: antimicrobial susceptibility test
BATSI: boronic acid transition state inhibitor
NCF: nitrocefin
CEF: cephalothin
CTX: cefotaxime
FEP: cefepime
FDC: cefiderocol
TOL: ceftolozane
CAZ: ceftazidime
MOPS: 3-(*N*-morpholino)propanesulfonic acid
MIC: minimum inhibitory concentration
DCM: dichloromethane
TFA: trifluoroacetic acid
Et_2_O: diethylether
THF: tetrahydrofuran
