# Supplementary material for: Synthesis of a Novel Boronic Acid Transition State Inhibitor, MB076: A Heterocyclic Triazole Effectively Inhibits Acinetobacter-Derived Cephalosporinase Variants with an Expanded-Substrate Spectrum
Source: J Med Chem. 2023 Jun 26;66(13):8510–25. doi: 10.1021/acs.jmedchem.3c00144 (PMC10350917; doi:10.1021/acs.jmedchem.3c00144)
Supplement: Supplementary file 1 — jm3c00144_si_001.pdf [file jm3c00144_si_001.pdf]

Supporting Information

Synthesis of a novel boronic acid transition state inhibitor (BATSI), MB076: a heterocyclic triazole effectively inhibits *Acinetobacter*-derived cephalosporinase (ADC) variants with an expanded-substrate spectrum.

Rachel A. Powers[1], Cynthia M. June[1], Micah C. Fernando[1], Erin R. Fish[1], Olivia L. Maurer[1], Rachelle M. Baumann[1], Trevor J. Beardsley[1], Magdalena A. Taracila[2,3], Susan D. Rudin[2,3], Kristine M. Hujer[2,3], Andrea M. Hujer[2,3], Nicolò Santi[5], Valentina Villamil[5], Maria Luisa Introvigne[5], Fabio Prati[5], Emilia Caselli[5*], Robert A. Bonomo[2,3,4*], Bradley J. Wallar[1*]*

1. Department of Chemistry, Grand Valley State University, Allendale, MI, USA 49401;
2. Department of Medicine, Case Western Reserve University School of Medicine, Cleveland, OH, USA 44106;
3. Research Service, Louis Stokes Cleveland Department of Veterans Affairs Medical Center, Cleveland, OH, USA 44106;
4. Clinician Scientist Investigator, Louis Stokes Cleveland Department of Veterans Affairs Medical Center, Cleveland, OH, USA; Departments of Pharmacology, Molecular Biology and Microbiology, Biochemistry, and Proteomics and Bioinformatics, Case Western Reserve University School of Medicine, Cleveland, OH, USA; and CWRU-Cleveland VAMC Center for Antimicrobial Resistance and Epidemiology (Case VA CARES) Cleveland, OH, USA 44106;
5. Department of Life Sciences, University of Modena and Reggio Emilia, via Campi 103, 41125, Modena, Italy.

Corresponding authors:
powersra@gvsu.edu; wallarb@gvsu.edu; emilia.caselli@unimore.it; robert.bonomo@va.gov

Contents

Supplemental Figure 1. Purity determination by HPLC of **MB076** S2

Supplemental Figure 2. ^{1}H NMR and ^{13}C NMR of **MB076** S3

Supplemental Figure 3. Stability of compounds **S02030** and **MB076** in human plasma. S4

Supplemental Figure 4. Multiple alignment of ADCs -7, -30, -162, -212, -33, -219 using Clustal Omega. S5

Supplemental Figure 5. Overview of entire structure of ADC-7 (4U0T; B monomer) highlighting amino acid residues that differ from the other variants. S6

Supplemental Figure 6. Walleye stereoviews of the Polder omit maps for the ADC variant complexes with **MB076**. S7-S9

Supplemental Table 1. Crystallographic statistics of ADC apo and **MB076** complexes S10-S11

Supplemental Figure 1. Purity of Final Compound MB076 used for biochemical and biological studies.

The purity of compound **MB076** was tested at a concentration of 1 mg/mL ppm in DMSO/MeOH 1:9. The measurement was determined by analytical HPLC Agilent 1100 system, equipped with a quaternary pump, an autosampler, and an UV detector (Agilent). Chromatographic separation of a 10 µl sample injection was performed on a InfinityLab Poroshell 120 EC-C18 4.6 x 100 mm ID, 4 µm ps column (Agilent) at 40 °C and a 0.5 mL/min flow rate. A gradient elution scheme was used with mobile phase components, being 0.1% formic acid in water (A) and 0.1% formic acid in acetonitrile (B). The gradient started at 2% B which was maintained for 1 minute, then raised up to 98% B for 10 minutes and maintained for 5 further minutes. The gradient was decreased to 2% B in 0.1 minutes (6 seconds) and then kept to 2% B for 5 minutes to restore the starting conditions. UV-VIS analyzer was set to λ = 254 nm. Purity calculations were performed on the UV-VIS trace chromatogram.

MB076 eluted at 7.71 min with a purity of 99.2%.

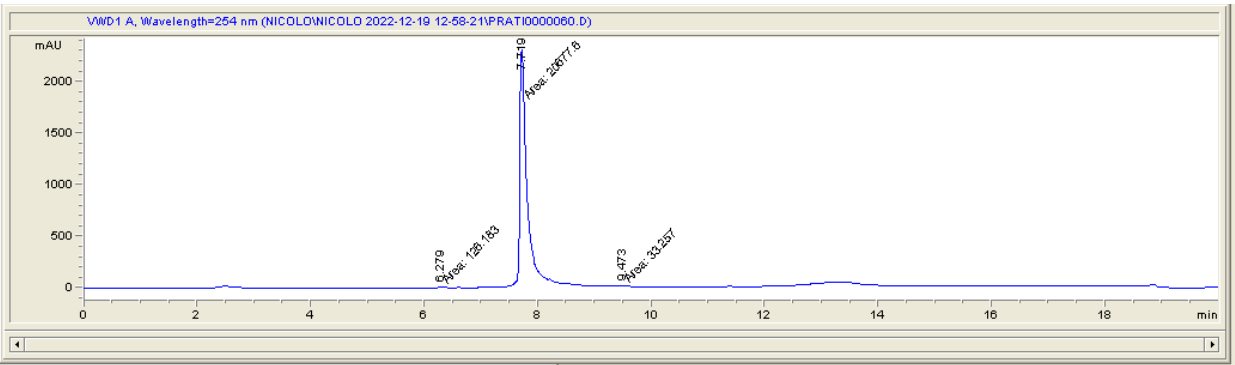

File Information	
LC-File	PRATI0000060.D
File Path	C:\CHEM32\1\DATA\NICOLO\NICOLO 2022-12-19 12-58-21\
Date	19-Dec-22, 16:14:06
Sample	MB076
Sample Info	
Barcode	
Operator	
Method	NICOLO.M
Analysis Time	19.996 min
Sampling Rate	0.0049 min (0.294 sec), 4121 datapoints

#	Time	Area	Height	Width	Area%	Symmetry
1	6.279	126.2	16.8	0.1255	0.606	1.594
2	7.719	20677.6	2299.3	0.1499	99.235	0.46
3	9.473	33.3	7.6	0.0726	0.160	0.786

Supplemental Figure 2. ^{1}H NMR and ^{13}C NMR of **MB076**

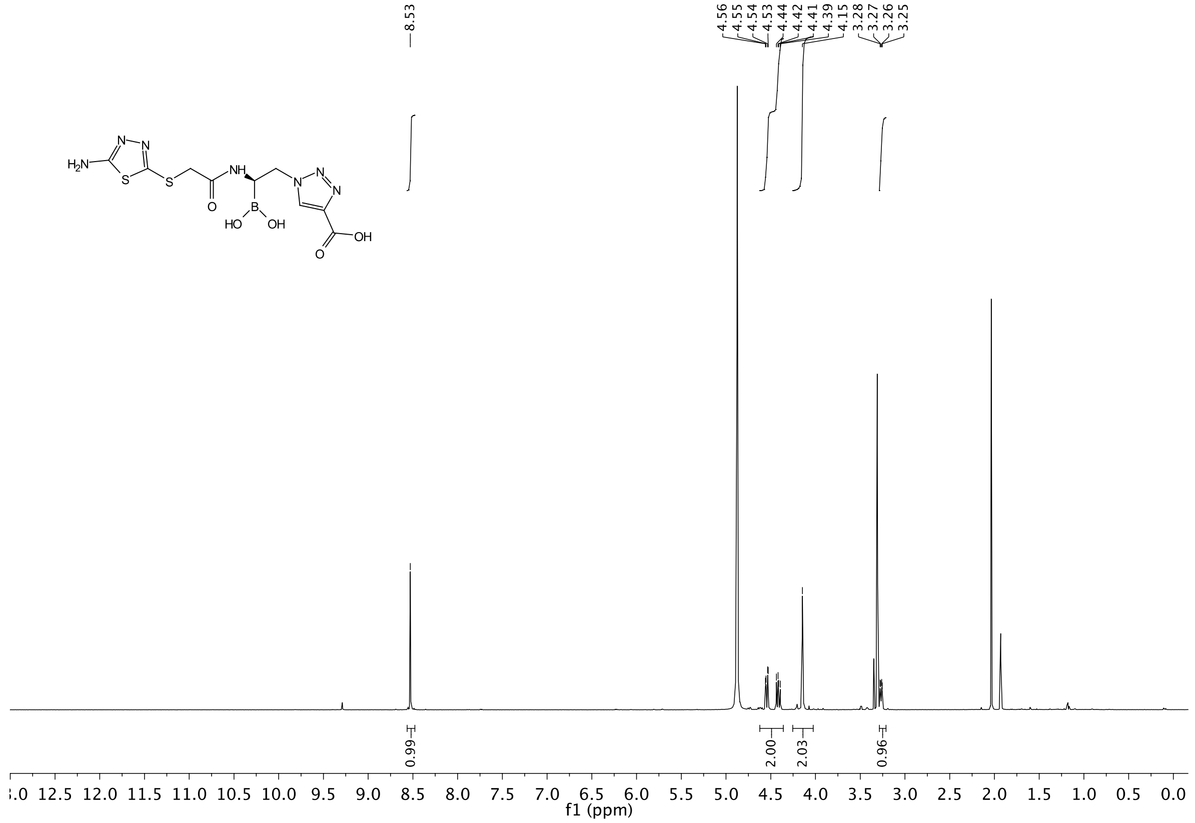

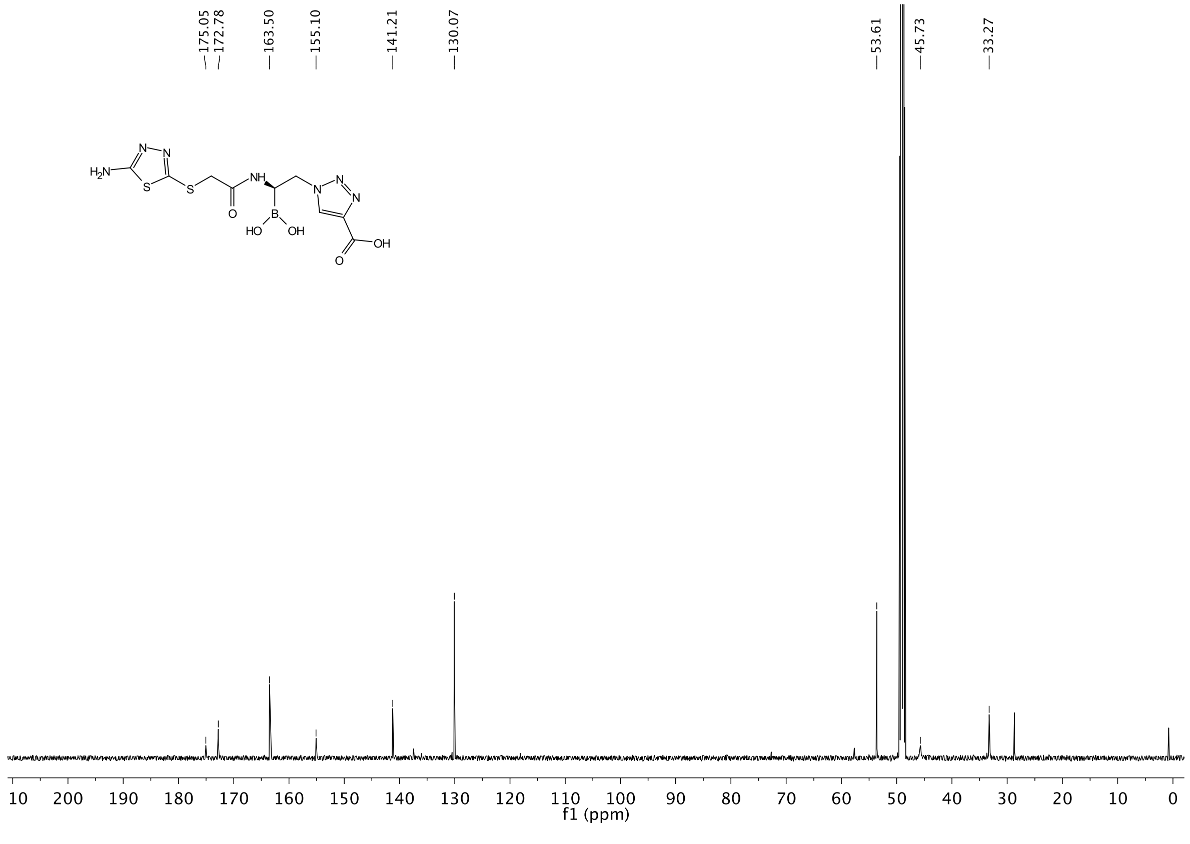

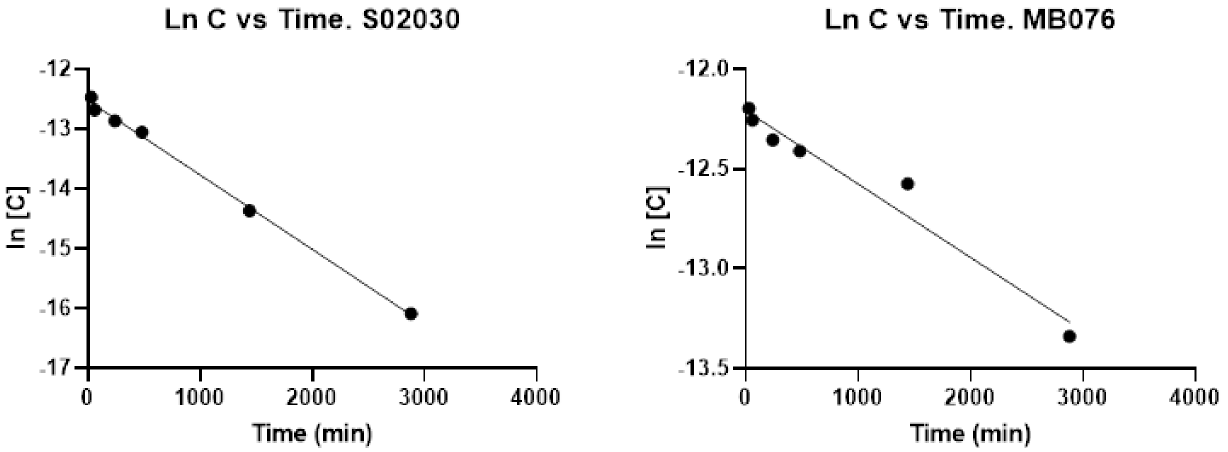

Supplemental Figure 3: Stability of compounds **S02030** and **MB076** in human plasma. The plots indicate the natural logarithm (ln) of the remaining compound concentration (C) versus incubation time.

```
ADC-7   MDNTPKDQEIKKLVDQNFKPLLEKYDVPGMAVGVIQNNKKYEMYYGLQSVQDKKAVNSNTIFELGSVSKLFTATAGGYAK 80
ADC-30  MGNTPKDQEIKKLVDQNFKPLLEKYDVPGMAVGVIQNNKKYEMYYGLQSVQDKKAVNSSTIFELGSVSKLFTATAGGYAK 80
ADC-162 MGNTPKDQEIKKLVDQNFKPLLEKYDVPGMAVGVIQNNKKYEMYYGLQSVQDKKAVNSSTIFELGSVSKLFTATAGGYAK 80
ADC-212 MGNTPKDQEIKKLVDQNFKPLLEKYDVPGMAVGVIQNNKKYEMYYGLQSVQDKKAVNSSTIFELGSVSKLFTATAGGYAK 80
ADC-33  MGNTPKDQEIKKLVDQNFKPLLEKYDVPGMAVGVIQNNKKYEMYYGLQSVQDKKAVNSSTIFELGSVSKLFTATAGGYAK 80
ADC-219 MGNTPKDQEIKKLVDQNFKPLLEKYDVPGMAVGVIQNNKKYEMYYGLQSVQDKKAVNSSTIFELGSVSKLFTATAGGYAK 80

ADC-7   NKGKISFDDTPGKYWKELKNTPIDQVNLLQLATYTSGNLALQFPDEVQTDQQVLTFFKDWKPKNPIGEYRQYSNPSIGLF 160
ADC-30  NKGKISFDDTPGKYWKELKNTPIDQVNLLQLATYTSGNLALQFPDEVKTDQQVLTFFKDWKPKNSIGEYRQYSNPSIGLF 160
ADC-162 NKGKISFDDTPGKYWKELKNTPIDQVNLLQLATYTSGNLALQFPDEVKTDQQVLTFFKDWKPKNSIGEYRQYSNPSIGLF 160
ADC-212 NKGKISFDDTPGKYWKELKNTPIDQVNLLQLATYTSGNLALQFPDEVKTDQQVLTFFKDWKPKNSIGEYRQYSNPSIGLF 160
ADC-33  NKGKISFDDTPGKYWKELKNTPIDQVNLLQLATYTSGNLALQFPDEVKTDQQVLTFFKDWKPKNSIGEYRQYSNPSIGLF 160
ADC-219 NKGKISFDDTPGKYWKELKNTPIDQVNLLQLATYTSGNLALQFPDEVKTDQQVLTFFKDWKPKNSIGEYRQYSNPSIGLF 160

ADC-7   GKVVALSMNKPFDQVLEKTIFPALGLKHSYVNVPKTQMQNYAFGYNQENQPIRVNPGPLDA-PAYGVKSTLPDMLSFIHA 240
ADC-30  GKVVALSMNKPFDQVLEKTIFPALGLKHSYVNVPKTQMQNYAFGYNQENQPIRVNPGPLDA-PAYGVKSTLPDMLSFIHA 240
ADC-162 GKVVALSMNKPFDQVLEKTIFPALGLKHSYVNVPKTQMQNYAFGYNQENQPIRVNPGPLDA-PEYGVKSTLPDMLSFIHA 240
ADC-212 GKVVALSMNKPFDQVLEKTIFPALGLKHSYVNVPKTQMQNYDFGYNQENQPIRVNPGPLDALAAYGVKSTLPDMLSFIHA 240
ADC-33  GKVVALSMNKPFDQVLEKTIFPALGLKHSYVNVPKTQMQNYAFGYNQENQPIRVNRGPLDAAPAYGVKSTLPDMLSFIHA 240
ADC-219 GKVVALSMNKPFDQVLEKTIFPALGLKHSYVNVPKTQMQNYAFGYNQENQPIRVNRGPLDAAPAYDVKSTLPDMLSFIHA 240

ADC-7   NLNPQKYPTDIQRAINETHQGRYQVNTMYQALGWEEFSYPATLQTLLDSNSEQIVMKPNKVTAISKEPSVKMYHKTGSTS 320
ADC-30  NLNPQKYPADIQRAINETHQGRYQVNTMYQALGWEEFSYPATLQTLLDSNSEQIVMKPNKVTAISKEPSVKMYHKTGSTT 320
ADC-162 NLNPQKYPADIQRAINETHQGRYQVNTMYQALGWEEFSYPATLQTLLDSNSEQIVMKPNKVTAISKEPSVKMYHKTGSTT 320
ADC-212 NLNPQKYPADIQRAINETHQGRYQVNTMYQALGWEEFSYPATLQTLLDSNSEQIVMKPNKVTAISKEPSVKMYHKTGSTN 320
ADC-33  NLNPQKYPADIQRAINETHQGRYQVNTMYQALGWEEFSYPATLQTLLDSNSEQIVMKPNKVTAISKEPSVKMYHKTGSTN 320
ADC-219 NLNPQKYPADIQRAINETHQGRYQVNTMYQALGWEEFSYPATLQTLLDSNSEQIVMKPNKVTAISKEPSVKMYHKTGSTN 320

ADC-7   GFGTYVVFIPKENIGLVMLTNKRIPNEERIKAAYVVLNAIKK 361
ADC-30  GFGTYVVFIPKENIGLVMLTNKRIPNEERIKAAYAVLNAIKK 361
ADC-162 GFGTYVVFIPKENIGLVMLTNKRIPNEERIKAAYAVLNAIKK 361
ADC-212 GFGTYVVFIPKENIGLVMLTNKRIPNEERIKAAYAVLNAIKK 362
ADC-33  GFGTYVVFIPKENIGLVMLTNKRIPNEERIKAAYAVLNAIKK 362
ADC-219 GFGTYVVFIPKENIGLVMLTNKRIPNEERIKAAYAVLNAIKK 362
```

Supplemental Figure 4. Multiple alignment of ADCs -7, -30, -162, -212, -33, -219 using Clustal Omega. All ADC sequences are > 97% identical; without ADC-7, the rest are 99% identical.

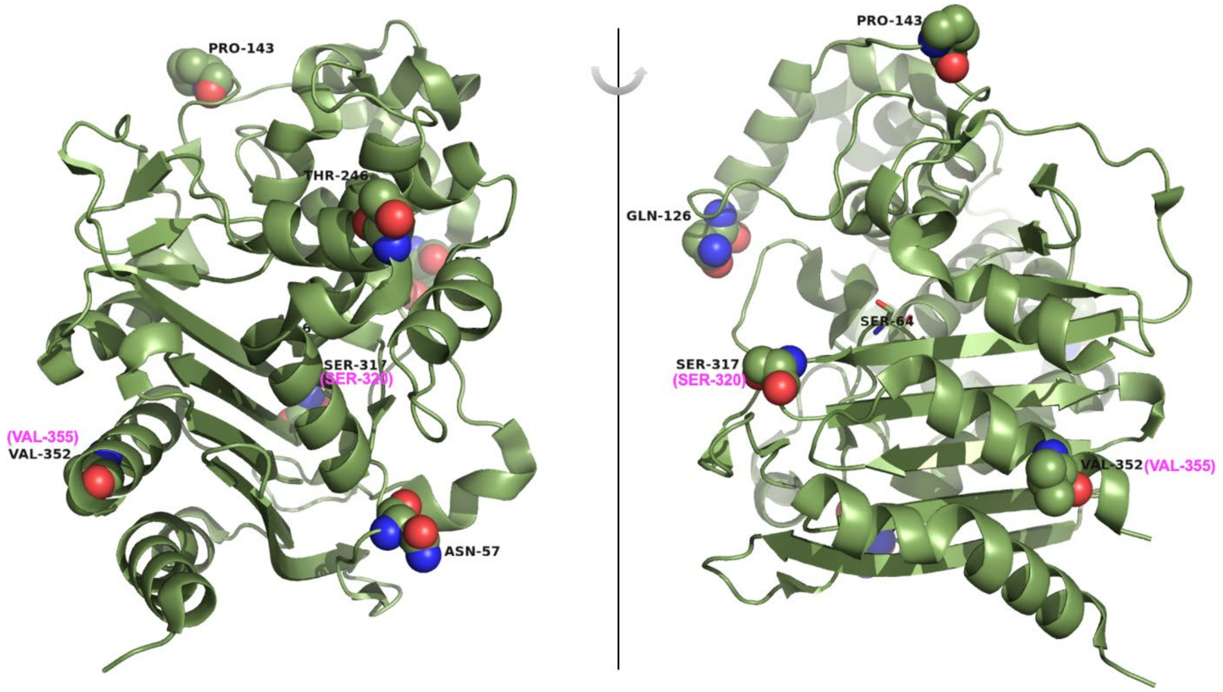

Supplemental Figure 5. Overview of entire structure of ADC-7 (4U0T; B monomer) highlighting amino acid residues that differ from the other variants. All differences occur on the surface of the enzyme. In previous ADC-7 structures, Ser320 (Thr or Asn in variants) is the only residue that has been observed to make interactions with ligands, via main chain (not side chain) atoms. Left and right panels differ by a 180 degree rotation around the vertical axis. Where SANC numbering differs from the PDB residue numbering, the SANC number is indicated in parentheses and magenta.

A. ADC-7/MB076 (contoured at 4.0 σ)

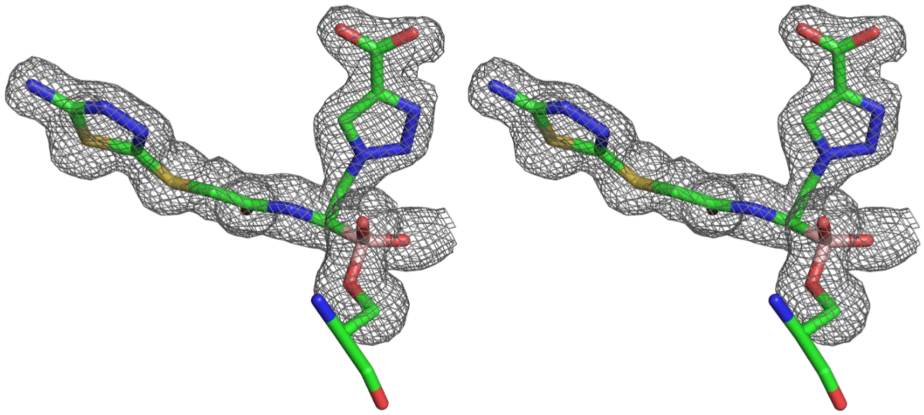

B. ADC-30/MB076 (contoured at 3.5 σ)

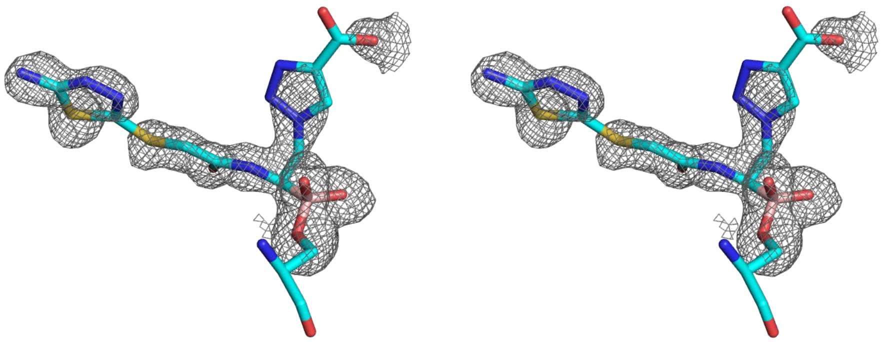

C. ADC-33/MB076 (contoured at 4.0 σ)

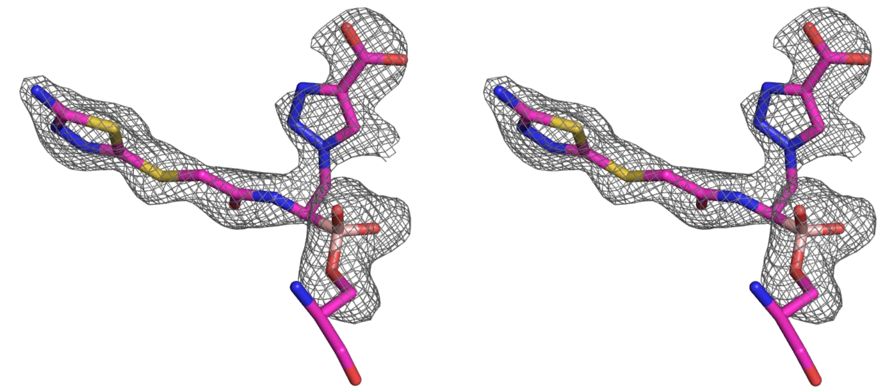

D. ADC-162/MB076 (contoured at 4.0 σ)

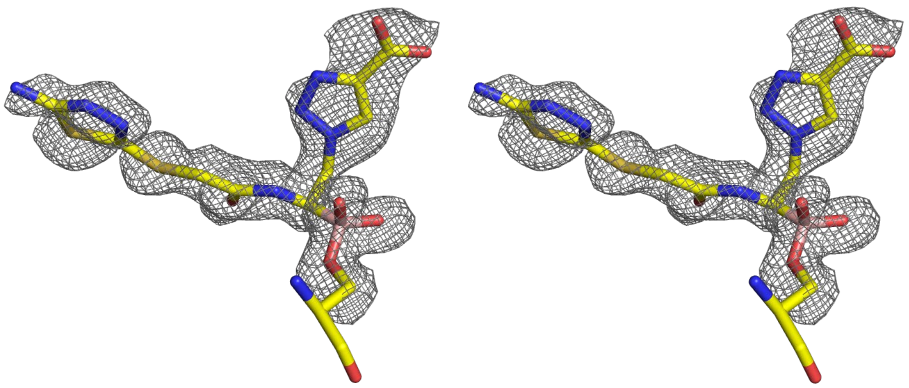

E. ADC-212/MB076 (contoured at 3.6 σ)

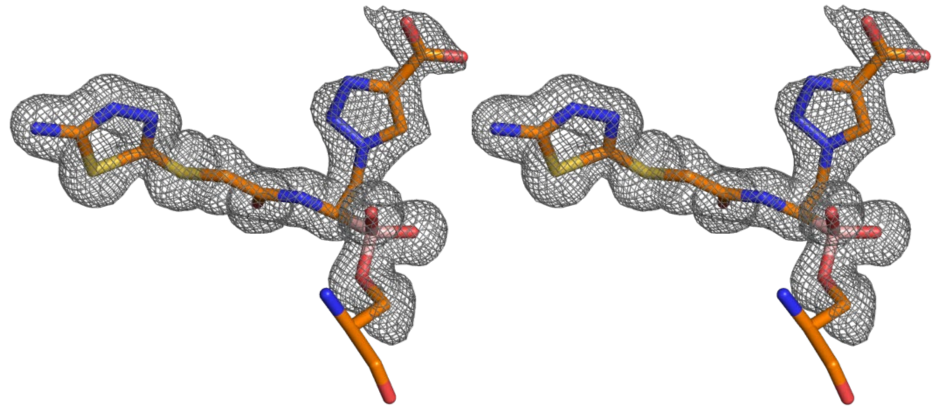

F. ADC-219/MB076 (contoured at 4.0 σ)

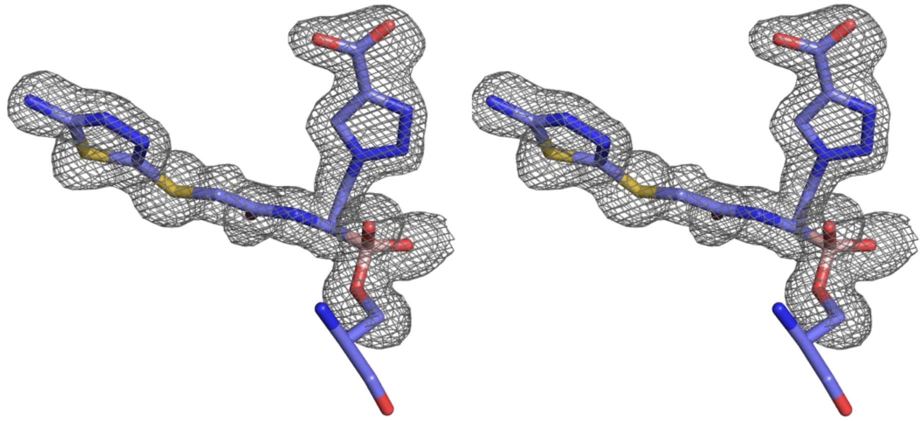

Supplemental Figure 6. Walleye stereoviews of the Polder omit maps for the ADC variant complexes with **MB076**. Maps were calculated with Phenix on the final refined models. The specific panel that shows each ADC variant are as follows: **A.** ADC-7/MB076 (contoured at 4.0 σ), **B.** ADC-30/MB076 (contoured at 3.5 σ), **C.** ADC-33/MB076 (contoured at 4.0 σ), **D.** ADC-162/MB076 (contoured at 4.0 σ), **E.** ADC-212/MB076 (contoured at 3.6 σ), **F.** ADC-219/MB076 (contoured at 4.0 σ).

Supplemental Table 1. Crystallographic statistics of ADC apo and MB076 complexes

	ADC-7 / MB076	ADC-30	ADC-30 / MB076	ADC-33	ADC-33 / MB076	ADC-162	ADC-162 / MB076
Cell constants (Å;°)	*a*=88.41, *b*=81.08<br>*c*=105.44<br>*α*=*γ*=90, *β*=113.66	*a*=43.15, *b*=83.49<br>*c*=205.33<br>*α*=*β*=*γ*=90	*a*=41.44, *b*=83.44<br>*c*=179.81<br>*α*=*β*=*γ*=90	*a*=41.12, *b*=83.55<br>*c*=205.59<br>*α*=*β*=*γ*=90	*a*=42.86, *b*=83.99<br>*c*=200.95<br>*α*=*β*=*γ*=90	*a*=43.05, *b*=83.63<br>*c*=204.34<br>*α*=*β*=*γ*=90	*a*=43.17, *b*=83.29<br>*c*=209.17<br>*α*=*β*=*γ*=90
Space group	P 2_1	P $2_12_12_1$	P $2_12_12_1$	P $2_12_12_1$	P $2_12_12_1$	P $2_12_12_1$	P $2_12_12_1$
Resolution (Å)	80.97-1.53<br>(1.66–1.53)[a]	77.34–1.48<br>(1.58–1.48)	89.91–1.59<br>(1.78–1.59)	77.40–1.25<br>(1.37–1.25)	100.48–1.83<br>(2.07-1.83)	77.40–1.41<br>(1.54–1.41)	77.38–1.48<br>(1.60–1.48)
Unique reflections	152952 (7647)	93283 (4664)	54143 (2708)	145507 (7274)	41360 (2069)	109317 (5467)	78568 (3828)
Total reflections	524823 (20880)	647438 (32624)	352856 (18956)	2002264 (104571)	243935 (12357)	646854 (28932)	395913 (18460)
R_{merge} (%)	5.0 (49.7)	8.6 (104.3)	7.7 (112.9)	12.2 (167.2)	22.8 (116.3)	7.8 (74.0)	7.3 (93.7)
Rpim (%) (all I + I-)	3.2(36.9)	3.5 (41.8)	3.2 (45.4)	3.4 (45.3)	10.2 (51.1)	3.5 (34.4)	3.4 (44.4)
CC(1/2)	0.999 (0.704)	0.999 (0.583)	0.999 (0.652)	0.997 (0.336)	0.990 (0.479)	0.998 (0.648)	0.999 (0.501)
Completeness (%)[b]	91.1 (47.7)	90.7 (58.6)	86.9 (57.9)	91.1 (55.6)	92.4 (53.1)	93.4 (50.6)	89.5 (65.5)
<I/σ>	11.9 (1.6)	11.7 (1.5)	13.1 (1.5)	12.2 (1.7)	5.9 (1.7)	10.8 (1.7)	12.0 (1.6)
Resolution for refinement (Å)	56.87–1.53	32.39–1.48	44.95–1.59	35.67–1.25	64.44–1.83	36.72–1.41	37.38-1.48
No. of protein residues	1427	714	711	718	717	714	714
No. of waters	1340	708	494	715	448	590	681
RMSD bond lengths (Å)	0.007	0.010	0.005	0.009	0.003	0.009	0.002
RMSD bond angles (°)	0.862	1.051	0.696	1.050	0.563	0.992	0.480
R-factor (%)	17.9	17.2	18.8	17.4	20.4	18.6	21.0
R_{free} (%)[c]	21.7	20.6	22.99	20.5	24.8	21.8	25.0
Average B factor, protein (Å²)	31.39	22.54	26.74	20.61	26.46	23.11	21.64
Average B factor, ligand (Å²)	31.03	n/a	32.14	n/a	30.69	n/a	26.78
Average B factor, waters (Å²)	35.46	29.13	30.54	30.36	29.03	28.72	26.77

[a] Values in parentheses are for the highest resolution shell.
[b] Fraction of theoretically possible reflections observed.
[c] R_{free} was calculated with 5% of reflections set aside randomly.

	ADC-212	ADC-212 / MB076	ADC-219	ADC-219 / MB076
Cell constants (Å;°)	*a*=49.39, *b*=69.81	*a*=43.48, *b*=71.82	*a*=43.04, *b*=83.57	*a*=45.52, *b*=172.44
	c=55.12	*c*=103.96	*c*=206.47	*c*=49.47
	$\alpha=\gamma=90$, $\beta=114.96$	$\alpha=\beta=\gamma=90$	$\alpha=\beta=\gamma=90$	$\alpha=\gamma=90$, $\beta=111.68$
Space group	$P\ 2_1$	$P\ 2_12_12_1$	$P\ 2_12_12_1$	$P\ 2_1$
Resolution (Å)	49.98-1.24	59.09-1.21	77.46-1.89	86.22-1.49
	(1.25- 1.24)	(1.33-1.21)	(1.90-1.89)	(1.65-1.49)
Unique reflections	83329 (941)	75449 (3772)	59691 (608)	81281 (4063)
Total reflections	297287 (3418)	551206 (17983)	372876 (4123)	323015 (15994)
R_{merge} (%)	2.9 (56.2)	5.2 (77.1)	9.8 (72.7)	10.6 (83.2)
Rpim (%) (all I + I-)	1.8 (34.8)	2.0 (38.3)	4.3 (30.3)	6.1 (47.8)
CC(1/2)	0.999 (0.741)	0.999 (0.682)	0.997 (0.885)	0.995 (0.595)
Completeness (%)[b]	87.4 (100.0)	95.0 (66.7)	98.1 (100.0)	92.0 (60.0)
$\langle I/\sigma \rangle$[a]	18.0 (2.1)	15.1 (1.5)	9.6 (2.2)	7.2 (1.5)
Resolution for refinement (Å)	49.98-1.24	31.21-1.21	38.73-1.89	28.74-1.49
No. of protein residues	355	355	706	717
No. of waters	360	359	369	724
RMSD bond lengths (Å)	0.007	0.012	0.006	0.006
RMSD bond angles (°)	0.961	1.214	0.791	0.885
R-factor (%)	15.8	17.0	19.6	17.4
R_{free} (%)[c]	17.7	19.3	24.2	20.9
Average B factor, protein ($Å^2$)	19.74	18.76	36.28	20.19
Average B factor, ligand ($Å^2$)	n/a	21.23	n/a	19.59
Average B factor, waters ($Å^2$)	29.83	26.78	37.53	27.68
